# Bioinspired Smart Nanogels for Rapid Blue Laser‐Activated Hemostasis in Gastrointestinal Bleeding

**DOI:** 10.1002/adma.202506955

**Published:** 2025-06-04

**Authors:** Ruijue Dan, Yu Huang, Shali Tan, Kaige Xu, Yuchen Zhang, Zhaohui Luan, Paipai Qi, Xingyue Zhang, Donghui Zhu, Wen Zhong, Chaoqiang Fan, Malcolm Xing, Shiming Yang

**Affiliations:** ^1^ Department of Gastroenterology Xinqiao Hospital NO.183, Xinqiao Street Chongqing 400037 China; ^2^ Department of Biomedical Engineering Stony Brook University Stony Brook NY 11794 USA; ^3^ School of Medicine & Nursing and First Affiliated Hospital Huzhou University Huzhou 313000 China; ^4^ Department of Mechanical Engineering University of Manitoba Winnipeg Manitoba R3T 2N2 Canada; ^5^ Department of Biosystems Engineering University of Manitoba Winnipeg Manitoba R3T 2N2 Canada

**Keywords:** bioinspired nanogel, blue laser endoscope, cirrhotic coagulation disorder, gastrointestinal bleeding, hemostasis, thrombin

## Abstract

Gastrointestinal bleeding (GIB) is a critical condition that requires rapid and effective intervention. Although thrombin is a widely used hemostatic agent, its efficacy is limited in the harsh environment of the digestive tract, especially in patients with chronic liver disease or coagulation disorders. Current treatment techniques often fall short, particularly when faced with severe blood loss and coagulation challenges. Here, a novel solution: waxberry‐inspired smart nanogels that offer a cost‐effective, highly efficient, and mechanically stable approach for local hemostasis is presented. Drawing inspiration from the microfibrous structures of waxberry, a waxberry‐like nano‐silica with a radially fibrous structure is synthesized for effective thrombin loading and release upon emergency. This nano‐silica, coated with GelMA, forms a stable nanogel network activated by blue laser during endoscopy. Within just 5 s, the nanogel effectively triggers coagulation, even in patients with coagulation disorders. The formed blood clots are stable enough to withstand the challenging conditions of the digestive tract, preventing secondary bleeding. Upon injection, thrombin rapidly converts fibrinogen to fibrin, creating a secondary network that reinforces clot stability. This dual‐network system demonstrates strong adhesive properties and effective hemostasis in the blood of cirrhotic patients, as well as in gastrointestinal bleeding scenarios involving the esophagus, stomach, and duodenum of mini‐pigs.

## Graphical abstract

Waxberry‐inspired branched‐silica nanogel can swiftly stop bleeding upon blue laser endoscopy for gastrointestinal hemostasis in the esophagus, stomach, and duodenum of mini‐pigs.

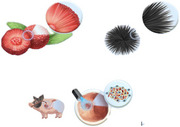

## 1 Introduction

Gastrointestinal bleeding (GIB) is a common clinical emergency requiring immediate attention.^[^ ^1^, ^2^, ^3^ ^]^ If left uncontrolled, hemorrhage from the gastrointestinal tract can be life‐threatening, with mortality rates ranging from 2% to 10%.^[^ ^4^, ^5^ ^]^ Rapid and effective hemostasis, along with preventing recurrent bleeding, is crucial to stabilize patients and save lives.^[^ ^6^ ^]^

Current primary treatments for GIB include through‐the‐scope hemoclips, thermal coagulation, or thrombin (Thr) therapy.^[^ ^7^, ^8^ ^]^ Hemoclips and thermal coagulation are popular for many scenarios due to their ability to provide physical hemostasis. However, these approaches have significant limitations when dealing with deep bleeding lesions, irregularly shaped wounds, diffuse bleeding, or hard‐to‐reach areas. Moreover, they often require cumbersome technical skills, an experienced operator, and present risks of tissue damage, rebleeding, or even perforation.^[^ ^8^, ^9^, ^10^ ^]^ In contrast, thrombin offers a straightforward and non‐invasive approach, easily applied to the bleeding site via endoscopy without causing damage to adjacent tissues or interfering with normal hemostatic functions.^[^ ^11^ ^]^ As a serine protease, thrombin catalyzes the conversion of fibrinogen into fibrin, forming an intricate network of proteins that effectively traps cellular components and stops bleeding.^[^ ^12^, ^13^ ^]^

Despite its effectiveness, thrombin‐based therapies for GIB face challenges, particularly in the complex gastrointestinal environment. The therapeutic efficacy is substantially compromised by three principal factors: 1) insufficient retention of thrombin at hemorrhagic sites, 2) rapid enzymatic inactivation of thrombin under harsh conditions, and 3) compromised structural instability of thrombin‐induced clots, due to pH variations and the presence of digestive enzymes, limits the efficacy of current thrombin treatments.^[^ ^14^, ^15^, ^16^ ^]^ Effective thrombin‐based hemostasis for GIB requires a system that (1) delivers and retains thrombin at the bleeding site, (2) ensures that the activity is preserved, and (3) the resultant blood clots resist degradation in the digestive tract, thereby minimizing the risk of recurrent bleeding.

Nature's diverse morphological structures often inspire innovative biomedical materials to address clinical challenges. For example, shark teeth inspired the design of microneedles with strong mechanical properties for drug delivery,^[^ ^17^ ^]^ and mussels have motivated the development of highly adhesive biomaterials.^[^ ^18^, ^19^ ^]^ Mushrooms inspired a gastric perforation occluder for repairing gastric damage.^[^ ^20^ ^]^ More recently, we took inspiration from the needle‐like protrusions of the waxberry, which efficiently transport moisture, nutrients, and sunlight while protecting the seed from microbial invasion, thus prolonging the fruit's shelf life.^[^ ^21^, ^22^, ^23^, ^24^ ^]^

Inspired by the waxberry's unique architecture, we developed dendritic mesoporous silica nanoparticles (DMSN) with branched morphologies for use as an efficient drug carrier. Compared to conventional mesoporous silica nanoparticles (MSN), DMSN features a 3D, branched porous structure with a high specific surface area suitable for loading substances like thrombin.^[^ ^25^, ^26^, ^27^, ^28^, ^29^ ^]^ These dendritic structures provide stable drug encapsulation and prevent degradation during biological transport, thereby preserving therapeutic efficacy.^[^ ^30^, ^31^, ^32^ ^]^ Additionally, the numerous branches of DMSN allow for functional modifications, which are beneficial for applications ranging from drug delivery to catalysis.^[^ ^33^, ^34^, ^35^, ^36^, ^37^, ^38^ ^]^

To enhance clot stability further, incorporating a “gel” network within the fibrin matrix can increase the adhesive energy, forming a more robust barrier against blood flow and digestive enzymes. Gelatin methacrylate (GelMA) has been widely used in biomedical applications due to its controllable gelation properties.^[^ ^39^ ^]^ In our prior work, we used lithium phenyl (2,4,6‐trimethylbenzoyl) phosphinate (LAP) as a photoinitiator and blue laser endoscopy (BLE) to trigger the photo‐crosslinking of GelMA, achieving effective hemostasis in GIB.^[^ ^40^, ^41^ ^]^ However, GelMA's thermosensitive properties require continuous heating to maintain fluidity, posing operational challenges for endoscopic procedures.^[^ ^42^, ^43^, ^44^ ^]^ To address this, we coated DMSN with GelMA, allowing the particles to remain stable and injectable without spontaneous gelation.

**Scheme 1:**
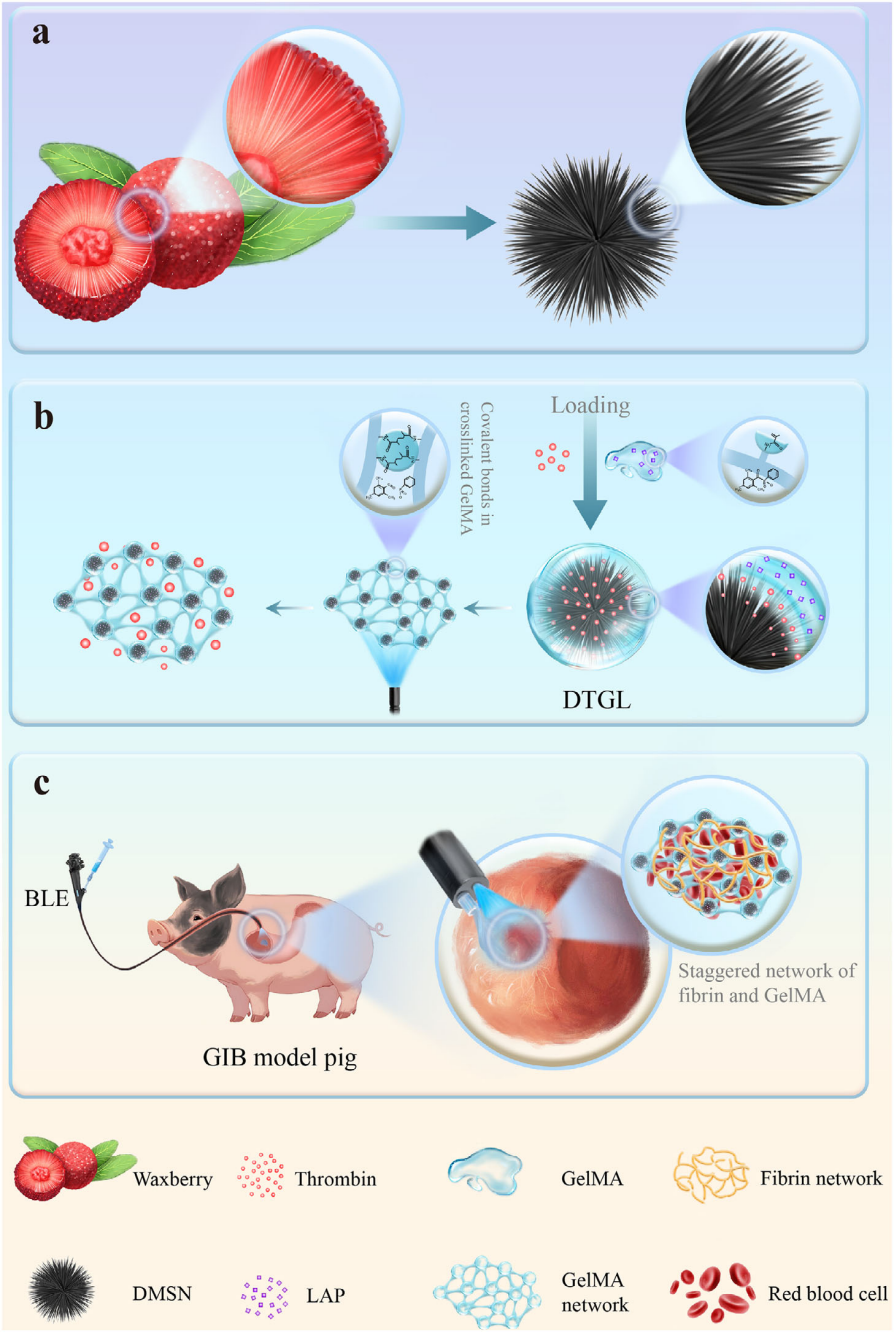
Schematic illustration of the synthesis and hemostatic performance of DTGL. (a) Waxberry‐inspired structure of the dendritic mesoporous silica nanoparticles (DMSN). (b) Fabrication of DTGL, where thrombin (Thr) is loaded into the nanopores, and Gelatin Methacrylate (GelMA) is coated onto the surface. GelMA network formation is triggered by blue laser irradiation. (c) Hemostatic efficacy of DTGL using blue laser endoscopy (BLE) in gastrointestinal bleeding (GIB) models.

In this study, we designed a novel hemostatic material (DMSN@Thr@Gel‐LAP, DTGL) inspired by waxberry's morphology. The material consists of thrombin‐loaded DMSN nanoparticles coated with GelMA and crosslinked using blue laser irradiation (Scheme 1a,b). The DMSN structure helps protect thrombin, while the macroporous network facilitates effective diffusion without compromising functionality. The blue laser triggers in‐situ GelMA gelation, forming a primary barrier that securely locks thrombin in place for subsequent fibrin network formation (Scheme 1b). This dual network not only accelerates hemostasis but also forms a denser clot structure. In vitro experiments demonstrated that our material rapidly promotes coagulation within 5 s, preserving thrombin activity and forming stable clots in complex digestive conditions. We further validated the hemostatic efficacy of this “gel‐protein” dual‐network system in mouse liver models and in the GIB of the esophagus, stomach, and duodenum of mini‐pigs (Scheme 1c).

## 2 Results and Discussion

### 2.1 Synthesis and Characterization of DTGL

**Figure 1:**
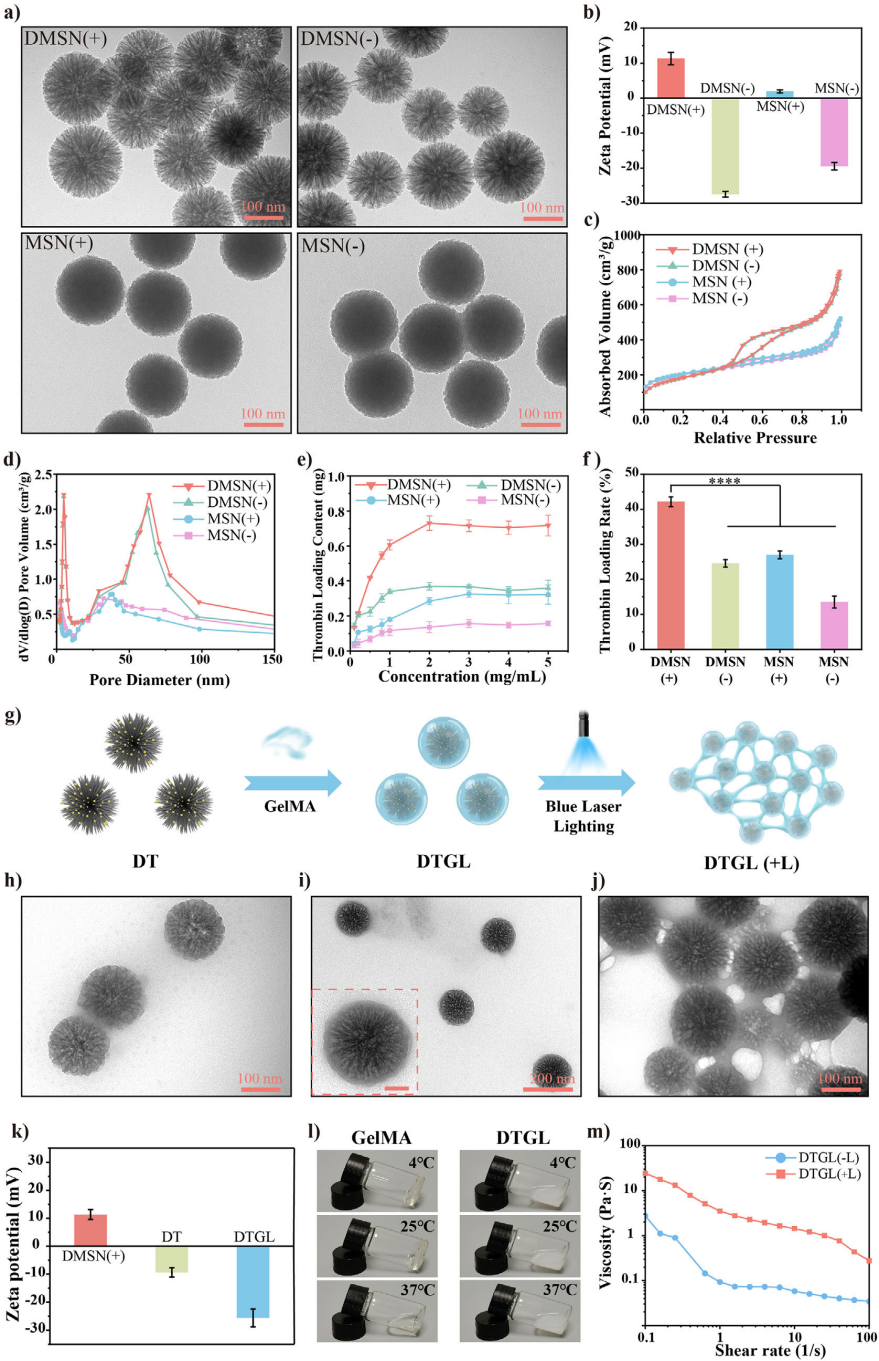
Preparation and characterizations of DTGL. (a) TEM images of DMSN (+), DMSN (‐), MSN (+), and MSN (‐). (b) Zeta potential measurements of DMSN (+), DMSN (‐), MSN (+), and MSN (‐), *n* = 3. (c) Nitrogen adsorption‐desorption isotherms. (d) Pore size distribution of DMSN (+), DMSN (‐), MSN (+), and MSN (‐). (e) Optimization of thrombin loading with DMSN at various mass ratios (*n* = 3, mean ± S.D.). (f) Loading efficiency of various samples (*n* = 3). (g) Schematic illustration of the preparation and gelation of DTGL (+). (h‐j) TEM images of DT (h), DTGL without blue laser irradiation (‐L) (i), and DTGL after blue laser irradiation (+L) (j). Insert image in (i), scale bar: 50 nm. (k) Zeta potential measurements of DMSN (+), DT, and DTGL (*n* = 3). (l) States of GelMA and DTGL at different temperatures. (m) Viscosity measurements of DTGL before (‐L) and after (+L) blue laser irradiation. (‐L): without irradiation; (+L): with irradiation. Data are expressed as mean ± S.D. ****p < 0.0001.

We initially synthesized dendritic mesoporous silica nanoparticles (DMSN) inspired by the waxberry structure (**Figure** **1**a). Since thrombin (Thr) is negatively charged,^[^ ^45^ ^]^ we synthesized DMSN and mesoporous silica nanoparticles (MSN) with similar sizes but different surface charges to investigate how their structures and charges impact Thr loading. Transmission electron microscopy (TEM) images revealed that the four nanoparticles had a consistent size of ≈150 nm. The zeta potential values for DMSN (+), DMSN (‐), MSN (+), and MSN (‐) were +11.3, −27.5, +1.9, and −19.4 mV respectively (Figure 1b).

To characterize the porosity of the synthesized nanoparticles, gas sorption analyses were conducted. Figure 1c shows the nitrogen sorption isotherms of the four samples, with detailed results summarized in Table S1 (Supporting Information). Based on the nitrogen sorption isotherms, DMSN (+) exhibited a mesoporous and macroporous structure. The pore size distribution was calculated using the non‐linear density functional theory model, as depicted in Figure 1d.

We next investigated the loading capacity of thrombin (Thr) in MSN (‐/+) and DMSN (‐/+). The loading capacity was examined by gradually increasing the input concentration of Thr. As shown in Figure 1e, the optimal loading amount was achieved at an input concentration of 2 mg/mL, corresponding to a loading efficiency of 42.2 ± 1.4%. Further increases in Thr concentration beyond this point did not significantly enhance loading efficiency (Figure 1e). Using the same methodology, the maximum loading efficiencies for MSN (‐), MSN (+), and DMSN (‐) were determined to be 13.5 ± 1.7%, 27.0 ± 1.1%, and 24.6 ± 1.1%, respectively, which were notably lower compared to DMSN (+) (Figure 1f). This difference was likely due to the higher positive surface charge density of DMSN (+), resulting from amino grafting, which allowed DMSN (+) to electrostatically adsorb Thr while effectively utilizing its high porosity. Additionally, Thr‐based injectable hemostats pose a risk of introducing strong coagulation activators into the circulatory system.^[^ ^46^, ^47^ ^]^ Gelatin methacryloyl (gelatin‐MA, hereafter signed as GelMA), however, can solidify, preventing the loss of biological material to unaffected areas. According to our previous research, GelMA can be photocrosslinked using lithium phenyl (2,4,6‐trimethylbenzoyl) phosphinate (LAP) as the photoinitiator, with blue laser endoscopy (BLE) acting as a reliable light source.^[^ ^40^, ^41^ ^]^ Therefore, to enable hemostats nanoparticles to seal wounds similarly to a spider web, we integrated GelMA and LAP into DT (resulting in DTGL), allowing the nanoparticles to form a network structure through photo‐crosslinking under blue laser light. As schemed in Figure 1g, GelMA was modified onto the surface of DT via electrostatic adsorption, forming the “core‐shell” structured DTGL. Upon exposure to blue laser irradiation, interparticle gel networks are formed within the DTGL system. To further validate our hypothesis, we employed TEM to observe the microprocesses step by step. As depicted in Figure 1h, the morphology and structure of DT, at its maximum Thr loading capacity, revealing that Thr was distributed within the hierarchical pores. As anticipated, the modified DTGL nanoparticles displayed a monodisperse “core–shell” structure (Figure 1i). As shown in Figure 1j, after 30 s of blue laser irradiation, a gel meshwork formed between the DTGL nanoparticles due to crosslinking via acrylic groups.

The zeta potential of DMSN changed from +11.3 mV (DMSN (+)) to −9.3 mV (DT), and then to −25.6 mV (DTGL) in distilled water, verifying the construction of DTGL (Figure 1k). Importantly, the GelMA coating on the surface of nanoparticles to form nanogel particles differs significantly from pure GelMA or simply adding nanoparticles to a GelMA solution. The DTGL nanoparticles exhibited extended fluidity without undergoing spontaneous gelation, enhancing their endoscopic injectability. The thrombin utilized in this study necessitates low‐temperature storage to maintain its stability and functionality. However, DTGL demonstrates superior fluid retention properties, enabling immediate availability for application following low‐temperature retrieval without requiring additional pretreatment steps. This stands in contrast to GelMA systems, which invariably demand thermal preconditioning (typically 37 °C) to maintain liquid‐phase processability prior to administration. This requirement stems from GelMA's inherent thermoresponsive behavior, undergoing spontaneous gelation transitions at both refrigeration (4 °C) and ambient (25 °C) temperatures, thereby significantly compromising injectability performance (Figure 1l). The viscosity of DTGL was also measured before and after blue laser irradiation, with the results presented in Figure 1m. Based on the viscosity curves, both solutions exhibited shear‐thinning behavior, showing decreasing viscosity at higher shear rates, characteristic of non‐Newtonian fluids. After light exposure, the viscosity of the DTGL solution increased sharply, likely due to the transition from discrete particles to gel networks. Overall, DTGL demonstrated favorable viscosity and loading efficiency, making it a promising candidate for subsequent investigations.

### 2.2 In Vitro Hemostatic Performance of DTGL

**Figure 2:**
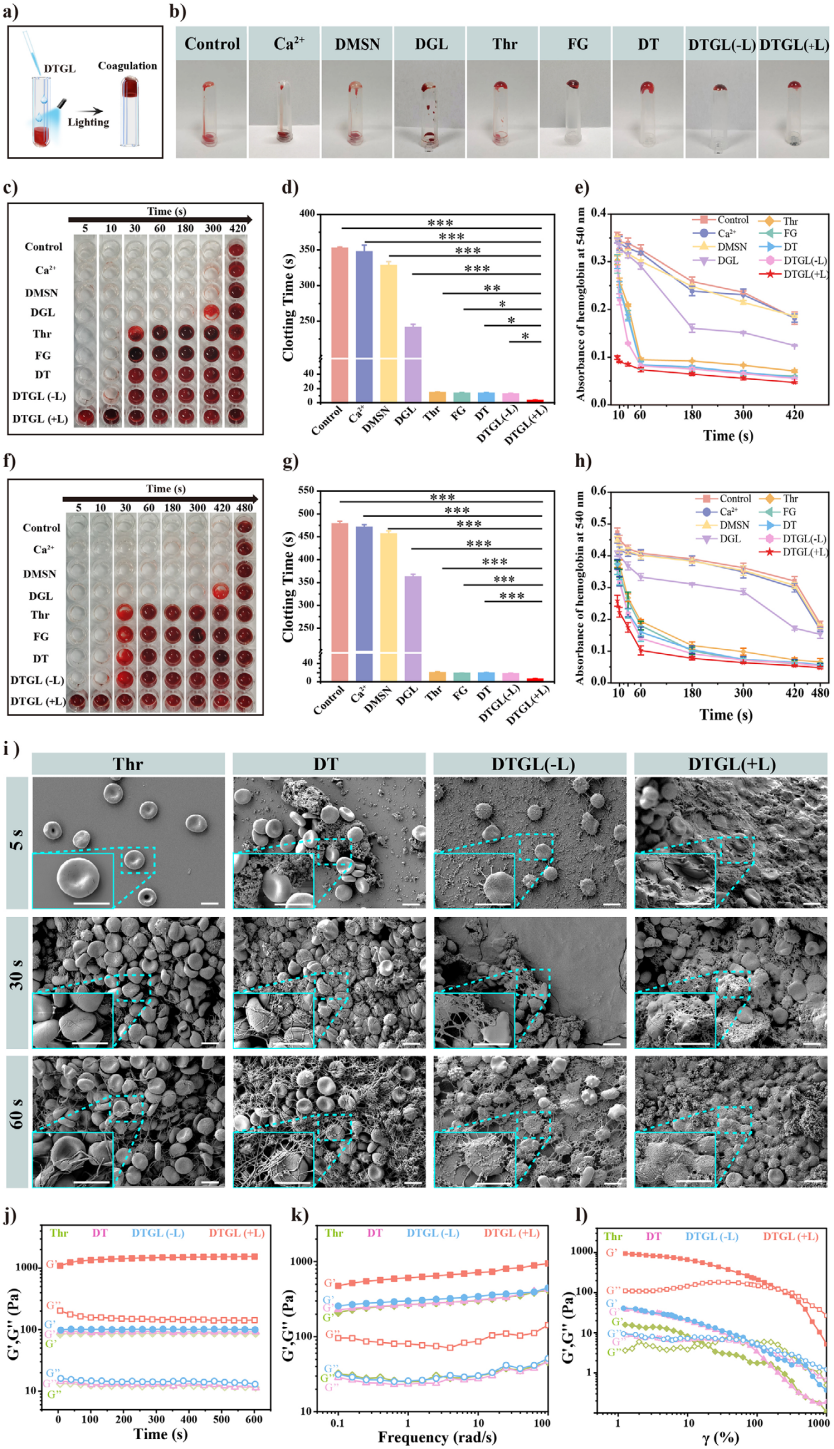
In vitro hemostatic performance and mechanism of DTGL. (a) Schematic illustration of the in vitro hemostatic performance of DTGL. (b) Photographs showing the blood coagulation performance for Control, Ca^2+^, DMSN, DGL, Thrombin (Thr), Fibrin glue (FG), DT, and DTGL groups. (‐L): without blue laser irradiation; (+L): with blue laser irradiation. (c‐d) Clot formation over time in blood samples from healthy volunteers. (e) Blood clotting kinetics over time for blood from healthy volunteers for Control, Ca^2+^, DMSN, DGL, Thr, FG, DT, and DTGL groups. (‐L): without blue laser irradiation; (+L): with blue laser irradiation. (*n* = 3). (f,g) Blood from cirrhotic patients for Control, Ca^2+^, DMSN, DGL, Thr, FG, DT, and DTGL groups. (‐L): without blue laser irradiation; (+L): with blue laser irradiation. (*n* = 5). (h) Blood clotting kinetics over time for blood from cirrhotic patients, for Control, Ca^2+^, DMSN, DGL, Thr, FG, DT, and DTGL groups. (‐L): without blue laser irradiation; (+L): with blue laser irradiation. (*n* = 3). (i) SEM images of samples taken at 5 s, 30 s, and 60 s post‐treatment. Scale bar: 5 µm. (j–l) Rheological performances of Thr, DT, and DTGL‐formed clots were systematically evaluated using dynamic time‐sweep tests (j), oscillation frequency tests (k), and strain amplitude sweep tests (l). (‐L): without blue laser irradiation; (+L): with blue laser irradiation. Data are expressed as mean ± S.D.; *p < 0.05, **p < 0.01, ***p < 0.001.

A successful hemostatic agent should be capable of promoting blood coagulation effectively. As depicted in **Figure** **2**a, the in vitro coagulation capacity of DTGL was initially evaluated by mixing it with anticoagulated human blood. As shown in Figure 2b, the control group, Ca^2+^ group, along with the DMSN and DGL groups, showed no thrombosis and maintained a smooth blood flow. In contrast, treatment with DT and DTGL (±L) resulted in complete coagulation of the blood in the tube, similar to the positive thrombin (Thr) group and fibrin glue (FG) group, indicating a remarkable hemostatic effect of DT and DTGL (±L), likely due to the presence of Thr.

To further investigate the hemostatic ability of DTGL, we monitored the clotting time of DTGL in contact with the blood of healthy volunteers using a 96‐well plate (Figure 2c). Under normal conditions, without any intervention, blood typically clots within 5 to 6 min.^[^ ^48^, ^49^, ^50^ ^]^ Consistent with expectations, both the Ca^2+^ and DMSN groups exhibited clotting time exceeding 300 s (>300 s), signifying negligible hemostatic activity. Upon hydrogel network establishment in the DGL cohort, a measurable reduction in clotting time was observed (241.1 ± 4.6 s). Positive control groups‐Thr and FG‐demonstrated hemostatic efficacy statistically equivalent to DT and DTGL (‐L), with respective clotting times of 14.2 ± 1.2 s, 13.4 ± 0.9 s, 13.5 ± 0.8 s, and 12.4 ± 1.1 s. In particular significance, the DTGL (+L) achieved a markedly accelerated clotting time (3.5 ± 0.5 s), demonstrating a statistically significant reduction compared to other groups (Figure 2d). We conducted comprehensive coagulation cascade analyses across all material groups (DMSN, DGL, Thr, FG, DT, DTGL(‐L), DTGL (+L)), performing activated partial thromboplastin time (APTT), prothrombin time (PT), and thrombin time (TT) assays with Ca^2+^‐containing samples serving as experimental controls.^[^ ^51^, ^52^ ^]^ As shown in Figure S1 (Supporting Information), the results demonstrated that the DTGL (+L) group exhibited notably shorter clotting times in all three assays with statistically significant differences compared to other experimental groups. We conducted quantitative analysis of erythrocyte adsorption from whole blood across all experimental groups, revealing that DTGL (+L) displayed the greatest erythrocyte adsorption capacity, with statistically significant differences observed when compared to control groups including Ca^2+^, DMSN, DGL, Thr, FG, and DTGL (‐L) (in the Figure S2a, Supporting Information). We additionally conducted quantitative analysis of platelet adsorption across all experimental groups, revealing that DTGL (+L) demonstrated significantly higher platelet adsorption capacity compared to other groups, including Ca^2+^, DMSN, DGL, Thr, FG, and DTGL(‐L) (in the Figure S2b, Supporting Information).^[^ ^51^, ^52^ ^]^ This suggests that the surface modification of nanoparticles with GelMA and subsequent photocrosslinking could significantly enhance the procoagulant effect.

To further substantiate the hemostatic efficacy of the DTGL nanoparticles, coagulation assays were also performed using blood samples from patients with decompensated liver cirrhosis (Figure 2f). Due to impaired liver function, achieving blood coagulation in these patients is typically challenging because of reduced synthesis of coagulation factors. Nevertheless, our experimental results demonstrated that DTGL (+L) maintained exceptional coagulation efficiency, as evidenced by rapid stable clot formation within 6.0 ± 1.1 s (Figure 2g). This performance was significantly superior to that of the Thr group (20.1 ± 2.0 s) and FG group (18.4 ± 1.0 s), also surpassed DT (19.1 ± 1.5 s) and DTGL (‐L) (17.5 ± 1.6 s) (Figure 2g).

Although DTGL has demonstrated a significant capacity for accelerating hemostasis, whether the gel network it forms can enhance the stability of blood clots remains to be further investigated. Therefore, we conducted coagulation kinetics experiments to compare the stability of clots formed by each group. In this experiment, clots formed at various time points were immersed in distilled water, and the absorbance of the resulting supernatant was measured at 540 nm to quantify clot dissolution. The results indicated that, whether formed by normal blood or blood from liver cirrhosis patients with deficient coagulation factors, the absorbance values were consistently lowest for the DTGL (+L) group compared to the other groups at each time interval, suggesting that DTGL (+L) induced the fastest and most robust clotting (Figure 2e,h).

To provide a more detailed analysis of these results, we further investigated the morphology of blood clots formed at different time points using scanning electron microscopy (SEM) (Figure 2i). At 5 s, in the Thr group, only scattered erythrocytes were observed without the formation of a fibrin network. Similarly, the DT group exhibited aggregation of nanoparticles around individual red blood cells but lacked a fibrin network. In the DTGL (‐L) group, fibrin strands were visible, emanating from the nanoparticles and partially surrounding dispersed red blood cells, but this interaction was insufficient to form a cohesive clot. In contrast, the DTGL (+L) group demonstrated a robust 3D “dual‐network” structure composed of interwoven nanogel networks and fibrin meshes that efficiently enveloped and aggregated multiple red blood cells, resulting in a stable clot within 5 s. By the 30 and 60 s time points, the fibrin networks in the Thr, DT, and DTGL (‐L) groups progressively densified as the coagulation process advanced. However, compared to these groups, the “dual‐network” structure in the DTGL (+L) group appeared more compact and structurally integrated, firmly encapsulating red blood cells (Figure 2i).

Additionally, we conducted a comparative analysis of the mechanical properties and viscosity of blood clots formed by Thr, DT, DTGL(‐L), and DTGL (+L) to further validate the superior stability of DTGL (+L)‐induced blood clots. Initially, rheological testing was performed to assess the enhancement of the mechanical properties of blood clots by DTGL following photo‐induced crosslinking. Dynamic time scanning and oscillation frequency tests revealed that, as every type of blood clots exhibited a solid‐gel state, the storage modulus (G′) was higher than the loss modulus (G″). However, the blood clots formed by DTGL (+L) demonstrated significantly greater stability, as evidenced by a much higher G′ compared to those formed by other groups (Figure 2j,k). Moreover, when the strain exceeded 20%, the Thr‐induced blood clots collapsed as the loss modulus (G″) surpassed the storage modulus (G′), whereas the DTGL (+L)‐induced blood clots remained intact until a strain of 252% (Figure 2l). This finding further confirmed that the dual‐network structure of DTGL's gel‐protein matrix significantly enhanced the stability of the blood clots. Simultaneously, viscosity testing was conducted on both types of blood clots, and the results showed that the viscosity of DTGL‐induced blood clots consistently exceeded that of other groups induced blood clots as the shear rate increased (Figure S3, Supporting Information). This demonstrated that the dual‐network structure not only improved the mechanical strength of the blood clots but also enhanced their adhesive properties under dynamic shear conditions. Overall, these results demonstrate that DTGL enhances the hemostatic efficiency of Thr and holds potential as a promising hemostatic agent.

### 2.3 Hemostatic Performance of DTGL In Mouse Liver

**Figure 3:**
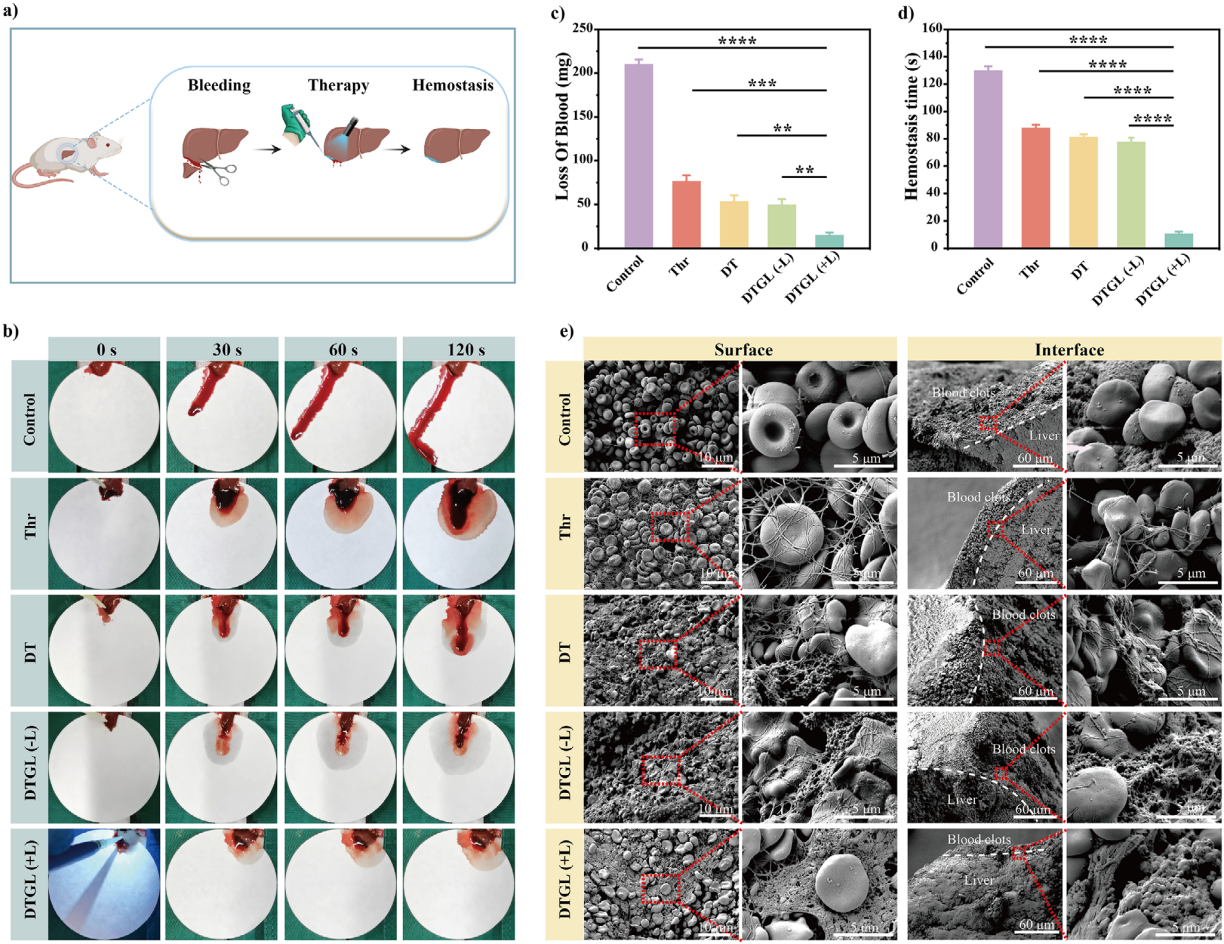
Hemostatic effect on hepatic lobectomy. (a) Schematic illustration of the hemostatic process. (b) Images showing untreated wounds (control group) and the hemostatic treatment process in severe liver wound models with Thrombin (Thr), DT, and DTGL. (‐L): without blue laser irradiation; (+L): with blue laser irradiation. (c) Blood loss measurements across treatment groups (*n* = 3) (d) Hemostasis time analysis (*n* = 3). (e) SEM images of the liver surface and the interface of sample‐blood clots after hemostasis. Data are expressed as mean ± S.D.; **p < 0.01, ***p < 0.001, ****p < 0.0001.

Mouse hepatic lobectomy models were established to quantitatively assess the in vivo hemostatic capability of DTGL. The experimental procedure is illustrated in **Figure** **3**a. After completely exposing the liver, one‐fourth of the liver lobe was excised to create a massive bleeding model. Hemostatic treatment was applied to the wound, and filter papers were placed beneath the liver to measure blood loss in different treatment groups. As shown in Figure 3b and Movie S1 (Supporting Information), the control group (without treatment) exhibited continuous bleeding from the wound site. In contrast, treatments with Thr, DT, and DTGL (‐L) resulted in temporary bleeding for a certain duration (Figure 3b and Movie S2–S4, Supporting Information). However, when treated with DTGL (+L), bleeding stopped immediately, and a clot formed rapidly, effectively sealing the wound (Figure 3b and Movie S5, Supporting Information).

Blood loss was then quantified by weighing the filter papers. As shown in Figure 3c, the control group had the highest blood loss, measuring 209.5 ± 6.2 mg. Blood loss for the Thr, DT, and DTGL (‐L) groups was 76.0 ± 7.4, 52.7 ± 7.7, and 49.0 ± 7.1 mg, respectively. Remarkably, DTGL (+L) exhibited excellent hemostatic properties, reducing blood loss to just 14.3 ± 3.8 mg‐approximately a 93.2% reduction compared to the control group.

Hemostatic time was also analyzed. The bleeding time at the liver injury site for DTGL (+L) was only 10.0 ± 2.2 s, while for the Control, Thr, DT, and DTGL (‐L) groups, it was 129.3 ± 3.7, 87.3 ± 2.9, 80.6 ± 2.6, and 77.0 ± 3.7 s, respectively (Figure 3d). These findings indicate that compared to Thr alone, DTGL (+L) demonstrated superior hemostatic performance, with a significantly shorter clotting time and reduced blood loss.

Wound tissues collected after different interventions were observed by SEM, as depicted in Figure 3e. In the control group, without treatment, minimal red blood cell aggregation and fibrin generation were observed, indicating only natural coagulation. Adding Thr enhanced coagulation, with fibrin networks forming around red blood cells. In the case of DTGL, GelMA served as an initial network, providing robust adhesion between the adhesive and the tissue, while Thr catalyzed the conversion of fibrinogen to fibrin, accelerating fibrin formation and establishing a physical barrier against bleeding.^[^ ^50^, ^53^ ^]^ This “gel‐protein” dual network effectively encapsulated red blood cells, forming stronger clots that adhered firmly to the wound surface. Hemostatic tests indicate that DTGL is an effective hemostatic agent for wound management following injury, demonstrating rapid coagulation and robust clot formation.

### 2.4 Protective Effect of DTGL on Thrombin Activity in a Simulated Gastrointestinal Environment

The gastrointestinal tract's dynamic pH variations and proteolytic environment severely compromise thrombin stability, leading to rapid enzymatic deactivation that limits therapeutic efficacy in digestive hemorrhage management. To address this critical challenge, we systematically evaluated the protective capacity of DTGL through comparative analyses of Thr, DT, and DTGL formulations under simulated gastrointestinal conditions. Enzymatic activity retention, thrombin‐antithrombin complex (TAT) quantification, and fibrinogen (FIB) consumption were monitored, with TAT levels positively correlating with active thrombin concentration and FIB surplus inversely reflecting enzymatic activity.

**Figure 4:**
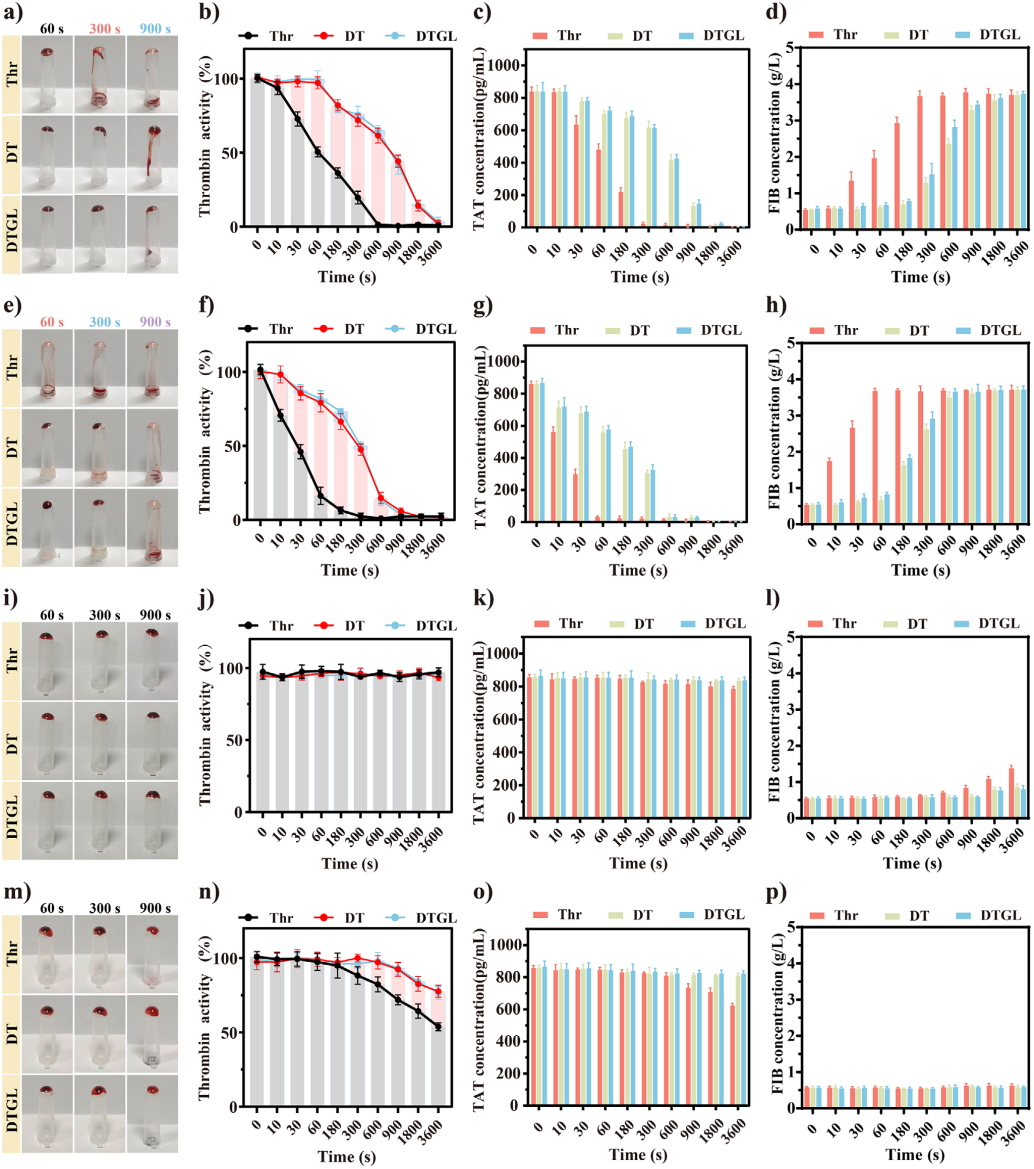
Effect of DTGL on thrombin activity in a simulated gastrointestinal environment. (a–d) Photographs (a), thrombin activity changes (b), TAT changes (c), and FIB changes (d) of Thr, DT, and DTGL in simulated gastric fluid (SGF) at 0, 10, 30, 60, 180, 300, 600, 900, 1800, and 3600 s (*n* = 3). (e‐h) Photographs (e), thrombin activity changes (f), TAT changes (g) and FIB changes (h) of Thr, DT, and DTGL in real gastric fluid (GF) at 0, 10, 30, 60, 180, 300, 600, 900, 1800, and 3600 s (*n* = 3). (i‐l) Photographs (i), thrombin activity changes (j), TAT changes (k), and FIB changes (l) of Thr, DT, and DTGL in simulated intestinal fluid (SIF) at 0, 10, 30, 60, 180, 300, 600, 900, 1800, and 3600 s (*n* = 3). (m‐p) Photographs (m), thrombin activity changes (n), TAT changes (o) and FIB changes (p) of Thr, DT, and DTGL in real intestinal fluid (IF) at 0, 10, 30, 60, 180, 300, 600, 900, 1800, and 3600 s (*n* = 3). Data are expressed as mean ± S.D.

Initially, the three groups of materials were pre‐treated with simulated gastric fluid (SGF, pH 0.9) for a certain period. As shown in **Figure** **4**a, Thr controls lost coagulation functionality within 300 s, while DT and DTGL maintained hemostatic capacity through 900 s (Figure 4a and Figure S4a, Supporting Information). Quantitative analysis revealed Thr's activity plummeted to 19.6% at 300 s, accompanied by depressed TAT (25.05 ± 5.84 pg/mL) and elevated FIB levels (3.67 ± 0.12 g/L). In contrast, DT and DTGL preserved 71.9% and 75.6% activity respectively, with corresponding TAT/FIB values of 44.25 ± 3.36 pg/mL/1.29 ± 0.12 g/L (DT) and 579.89 ± pg/mL/0.68 ± 0.05 g/L (DTGL) (Figure 4b–d). Under physiologically real gastric fluid (GF, pH 0.9 + pepsin), degradation accelerated markedly: Thr lost 83.8% activity within 60 s versus DT/DTGL retaining > 79% activity. Short exposure (60 s) caused almost Thr inactivation, while DT/DTGL maintained functionality through 300 s. Biochemical analyses confirmed this trend, with DT/DTGL showing 17‐fold higher TAT levels (561.69 ± pg/mL and 579.89 ± pg/mL) and 68% lower FIB concentrations than Thr controls at 60 s (Figure 4e–h and Figure S4b, Supporting Information).

Transitioning to intestinal conditions, simulated intestinal fluid (SIF) elicited comparable stability across the three groups (Figure 4i–l and Figure S5a, Supporting Information), which may be attributed to the SIF being a near‐neutral solution (pH 6.8) that has little effect on thrombin activity. However, enzyme‐contained intestinal fluid (IF) revealed differential inactivation kinetics: after 3600 s exposure, DT/DTGL maintained 77.6% and 76.3% activity versus Thr's 53.8%. Corresponding TAT concentrations (813.61 ± 10.16 pg/mL for DT, 826.30 pg/mL for DTGL vs 627.46 ± 9.01 pg/mL for Thr) and FIB levels (0.85 ± 0.07 g/L for DT, 0.80 ± 0.08 g/L for DTGL vs 1.38 g/L for Thr) further validated sustained bioactivity preservation (Figure 4m–p and Figure S5b, Supporting Information).

Overall, these results demonstrate that DT and DTGL can protect the activity of thrombin in the complex gastrointestinal environment. Although there is no significant difference between the two groups, it highlights that the comparable protection efficacy stems from their shared biomimetic DMSN architecture, inspired by unique acicular structure of waxberry. The dendritic macroporous channels of DMSN can effectively shield the loaded thrombin from the effects of the acidic alkaline conditions and digestive enzymes in the gastrointestinal tract, thereby preserving its activity.

### 2.5 Stability of DTGL Formed Blood Clots In Simulated Gastrointestinal Environment

Ensuring robust formation of a stable blood clot is essential for promoting hemostasis, preventing rebleeding, and facilitating wound healing, particularly within the dynamic and complex internal environment of living organisms. Therefore, we investigated the degradation of preformed clots induced by Thr, FG, DT, DTGL (‐L), and DTGL (+L) by quantifying the relative change in their mass and volume after submerging them in various digestive fluids for different periods.

**Figure 5:**
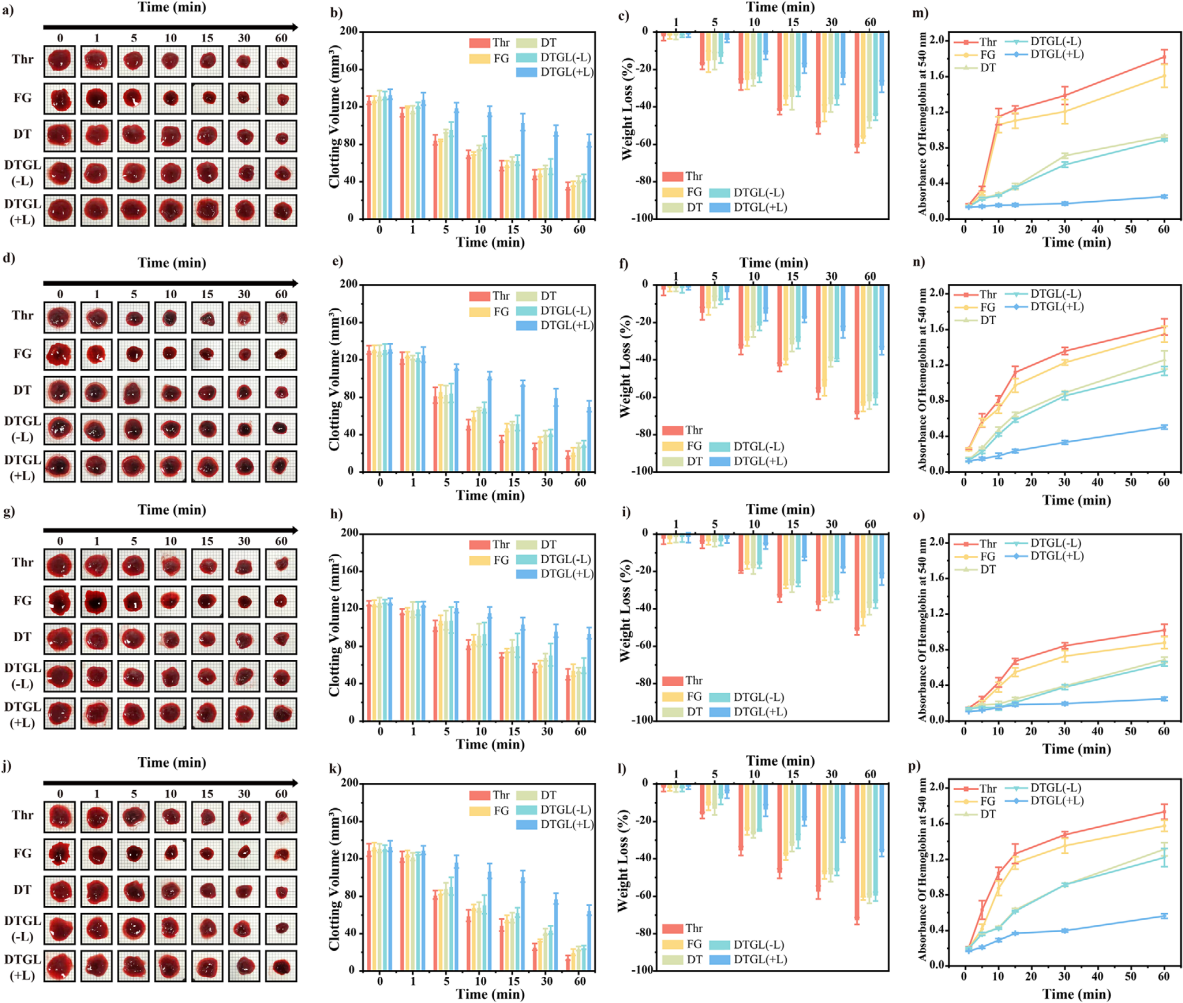
Stability of the blood clots formed by DTGL in digestive fluids. (a–c) Photographs (a), volume changes (b), and weight changes (c) of fresh blood clots in simulated gastric fluid (SGF) at 1, 5, 10, 15, 30, and 60 min (*n* = 3). (d–f) Photographs (d), volume changes (e), and weight changes (f) of fresh blood clots in real gastric fluid (GF) at 1, 5, 10, 15, 30, and 60 min (*n* = 3). (g–i) Photographs (g), volume changes (h), and weight changes (i) of fresh blood clots in simulated intestinal fluid (SIF) at 1, 5, 10, 15, 30, and 60 min (*n* = 3). (j–l) Photographs (j), volume changes (k), and weight changes (l) of fresh blood clots in real intestinal fluid at 1, 5, 10, 15, 30, and 60 min (*n* = 3). (m–p) Hemoglobin absorbance at 540 nm in SGF (m), SIF (n), real gastric fluid (o), and real intestinal fluid (p) over time (*n* = 3). Data are expressed as mean ± S.D.

First, clots formed from different groups were subjected to simulated gastric fluid (SGF) and real gastric fluid (GF). As illustrated in **Figure** **5**a, the blood clots formed by the DTGL (+L) group retained over 62.8 ± 4.9% of their residual volume and 70.9 ± 3.0% of their initial mass after incubation for 60 min, significantly higher compared to the other four groups (Figure 5a–c). Upon immersion in GF, a significant reduction in the volume and mass of the Thr‐induced blood clot was observed due to enzymatic degradation. However, clots formed by DTGL (+L) degraded significantly more slowly, retaining 53.2 ± 4.5% of their volume and 64.9 ± 2.2% of their mass after 60 min in real gastric fluid (Figure 5d–f). These values were significantly higher than those observed for both the FG group, DT group, and the DTGL (‐L) group. The enhanced stability of the clots in the DTGL (+L) group was attributed to the photocrosslinking of GelMA, which improved the long‐term stability of the clots, making them resistant to acidic and alkaline conditions‐crucial for reducing rebleeding in GIB.

To further investigate thrombus stability under intestinal conditions, we subjected thrombi generated by different formulations to both simulated intestinal fluid (SIF, pH 6.8) and physiologically real intestinal fluid (IF, pH 6.8) containing pancreatic enzymes. Similarly, when treated with SIF, the DTGL (+L) clots retained over 73.5 ± 4.8% of their volume, with a residual mass exceeding 75.9 ± 3.2% after 60 min (Figure 5g–i). Although the elevated pH of SIF attenuated the degradation of Thr‐induced clots, the remaining mass and volume were still significantly lower compared to those in the DTGL (+L) group. Consistent results were obtained with real intestinal fluid (IF), with a denser clot structure observed in the DTGL (+L) group compared to other groups, retaining approximately 50.3 ± 4.0% volume and 63.1 ± 1.7% mass after 60 min (Figure 5j–l).

These results indicate that the denser clots formed by DTGL (+L) consist of a tightly packed “gel‐protein” network, restricting the movement of digestive fluids, leading to a reduced lysis rate and rendering the clots more resistant to digestive fluid degradation and less prone to rupture.^[^ ^54^ ^]^

Simultaneously, the concentration of hemoglobin in the supernatants was measured using spectrophotometry at a wavelength of 540 nm. Higher concentrations of hemoglobin in the supernatant indicate fewer stable clots, as more red blood cells have lysed and released their hemoglobin. The Thr group exhibited the highest hemoglobin concentration, whereas the DTGL (+L) group demonstrated the lowest concentration, with the other two groups falling in between (Figure 5m–p). These findings are consistent with those in Figure 5a–l. Altogether, our results suggest that the blood clot formed by DTGL (+L) has enhanced stability against complex acid‐base and enzymatic degradation, supporting the potential application of DTGL (+L) in GIB management.

### 2.6 In Vivo Hemostatic Performance of DTGL in Swine Gastrointestinal Bleeding Models

**Figure 6:**
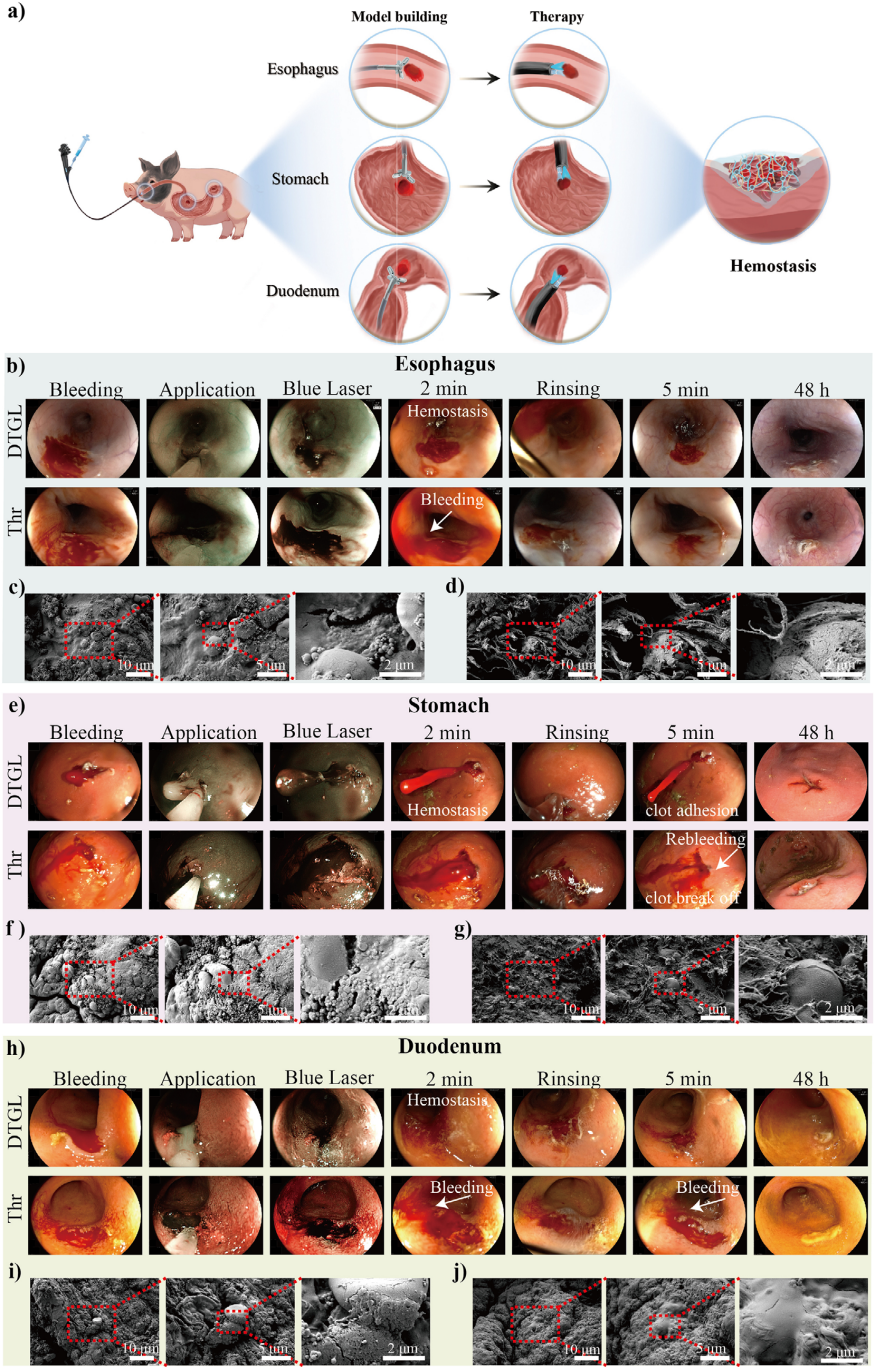
Hemostatic properties of DTGL in pig GIB model. (a) Schematic illustration of the hemostasis procedure and mechanism of DTGL under blue laser endoscopy (BLE) in pig GIB models. (b) Endoscopic images showing the esophageal hemostasis process of DTGL and Thrombin (Thr) under BLE. (c, d) SEM images of esophageal samples treated with DTGL (c) and Thr (d). (e) Endoscopic images of the gastric hemostasis process with DTGL and Thr under BLE. (f, g) SEM images of gastric samples treated with DTGL (f) and Thr (g). (h) Endoscopic images of the duodenal hemostasis process with DTGL and Thr under BLE. (i, j) SEM images of duodenal samples treated with DTGL (i) and Thr (j).

We demonstrated the rapid hemostatic performance of DTGL in a porcine gastrointestinal bleeding (GIB) model to further validate its in vivo efficacy in a clinically relevant setting (**Figure** **6**a). Under endoscopic guidance, GIB wounds were created by selectively targeting areas rich in blood vessels using biopsy forceps. After removing the esophageal wall and blood vessel, persistent bleeding was observed. The DTGL solution was immediately sprayed onto the bleeding site using a delivery catheter and exposed to blue laser endoscopy (BLE) for 30 s. As shown in Figure 6b and Movie S6 (Supporting Information), DTGL demonstrated immediate hemostatic efficacy, arresting hemorrhage within seconds with no further blood loss. After treatment, thorough irrigation with saline was performed, resulting in no disruption of the formed clot or rebleeding, indicating the stability of the clots.

In contrast, hemostasis using an equal volume of Thr for an esophageal hemorrhage failed to achieve effective clotting, with active bleeding observed throughout. After saline flushing, active bleeding persisted and gradually stopped after 5 min (Figure 6b, second row, and Movie S7, Supporting Information). Neither treatment group exhibited signs of irritability, melena, or hematemesis during the two‐day monitoring period. Endoscopic examination at 48 h post‐operation revealed only scab formation at the wound sites, with no signs of rebleeding.

The ineffectiveness of Thr was likely due to persistent bleeding and the difficulty in adhering to bleeding tissue surfaces because of lubricating mucus and the dynamic motility of the esophagus, which collectively impeded the secure attachment of the Thr‐generated clot. Conversely, DTGL produced a clot with enhanced adhesive properties, firmly bonding to the wound surface. To elucidate the hemostatic mechanism of DTGL, tissue samples containing hemostatic clots were examined by SEM, showing that DTGL adhered to the tissue surface, forming a confluent mass that wrapped red blood cells (Figure 6c). In contrast, the Thr group exhibited a visible fibrin matrix with aggregated erythrocytes (Figure 6d).

DTGL also showed superior in vivo hemostasis in a gastric bleeding model. Persistent brisk bleeding was promptly halted when DTGL was injected into the wound site, facilitating the formation of a stable columnar blood clot. DTGL adhered immediately to the gastric mucosa, creating a protective barrier that effectively retained the hemostatic agent at the injury site. No bleeding or clot dislodgement was observed after saline treatment at 5 min post‐application (Figure 6e and Movie S8, Supporting Information). However, in the Thr group, while clots initially formed, some disintegrated following saline irrigation, resulting in rebleeding (Figure 6e, second row, and Movie S9, Supporting Information). SEM analysis mirrored the findings in the esophagus, revealing that DTGL promoted a dense network structure that enclosed red blood cells and adhered tightly to the gastric tissue (Figure 6f). In contrast, the Thr group showed fibrin loosely wrapping the blood cells (Figure 6g).

The application of DTGL in the duodenum also confirmed its exceptional hemostatic capabilities. No rebleeding was observed during a 5‐min observation period and after saline irrigation. Gastroscopy revealed that the hydrogel adhered strongly to the duodenal wall and formed a hydrogel film (Figure 6h and Movie S10, Supporting Information). Similar to the esophagus observations, the Thr group exhibited persistent active bleeding and failed to achieve effective hemostasis (Figure 6h, second row, and Movie S11, Supporting Information). SEM results were consistent with previous findings (Figure 6i,j).

Following hemostasis of GIB using DTGL, all pigs survived without any signs of adverse reactions during the 48‐h observation period. The hemostatic capability of DTGL has been corroborated in cases of acute hemorrhage, including esophageal, gastric, and duodenal bleeding models. The exceptional performance of DTGL in achieving rapid hemostasis and maintaining clot integrity highlights its potential as a more reliable hemostatic agent. Its strong adhesion effectively sealed the hemorrhagic site, contributing to efficient hemostasis and preventing rebleeding.

### 2.7 Biocompatibility of DTGL

**Figure 7:**
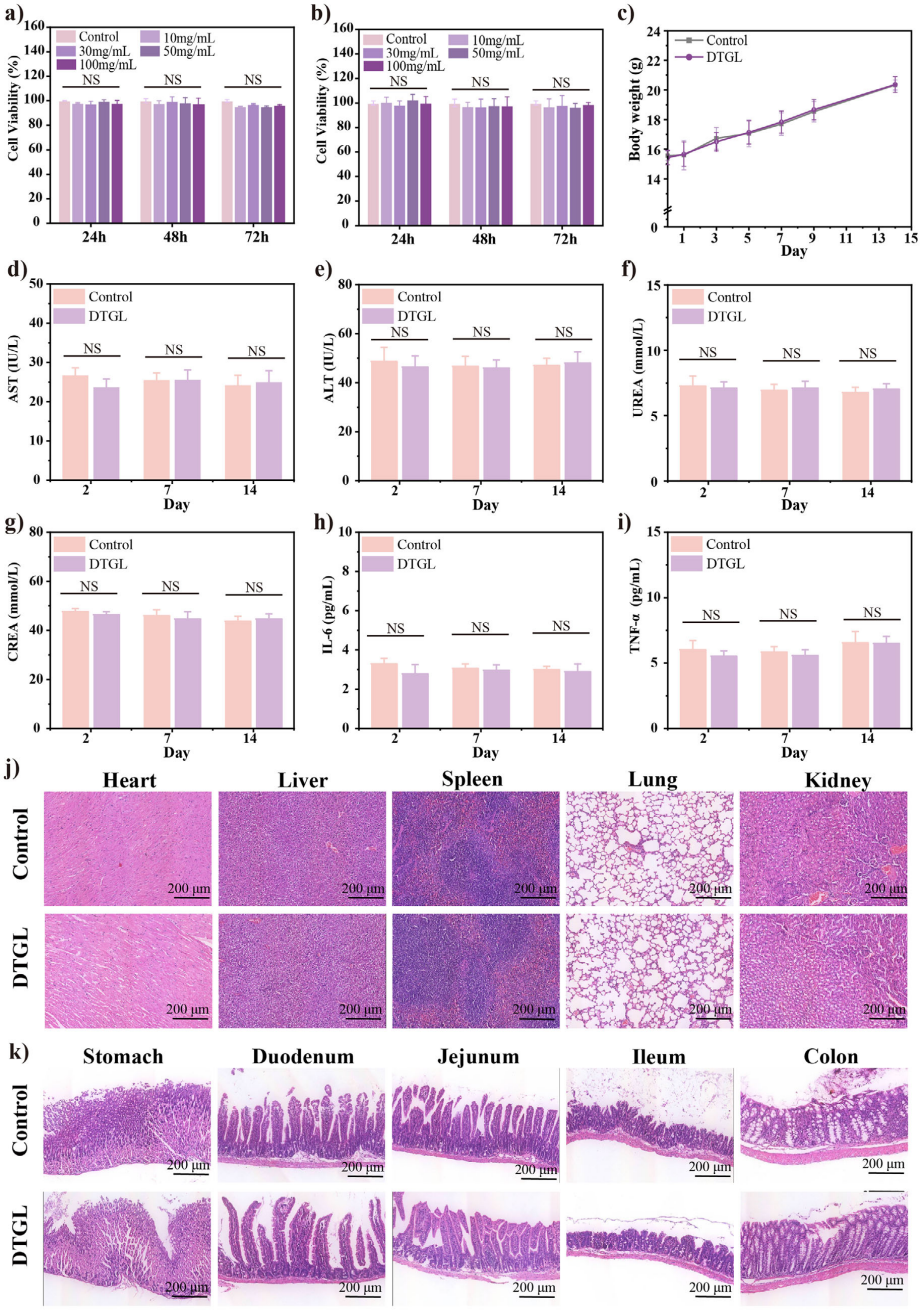
Biocompatibility assessments of the DTGL. (a, b) Cytotoxicity of different concentrations of DTGL (10, 30, 50, 100 mg/mL) on (a) GES‐1 and (b) HUVEC cells after 24, 48, and 72 h of culture (*n* = 3). (c) Weight distribution, and (d‐i) blood biochemical analysis of mice after oral administration of PBS and DTGL. (j) H&E staining of the major organ tissues (heart, liver, spleen, lung, kidney) on 14 day for intragastric administration. (k) H&E staining of the gastrointestinal tract (stomach, duodenum, jejunum, ileum, colon) on 14 day for intragastric administration. Data are presented as mean ± S.D.; NS, no significant difference.

Biocompatibility is a crucial consideration for in vivo GIB applications. GES‐1, HUVEC, and 3T3 cells were used to evaluate the in vitro cytocompatibility of DTGL. In the CCK‐8 assay, cell viability remained above 98%, with comparable growth observed after 1, 2, and 3 days of treatment, indicating that DTGL did not exhibit any toxic effects (**Figures** **7**a,b and S6, Supporting Information). Additionally, in vivo biocompatibility was assessed through intragastric administration of DTGL. During the treatment period, the toxicity profile of DTGL in mice was evaluated by monitoring changes in body weight. Notably, both the DTGL‐treated group and the PBS‐treated control group maintained stable body weights, with no discernible differences (Figure 7c). 2, 7, and 14 days post‐treatment, the mice were euthanized and serum samples were collected for biochemical analysis. No significant differences were observed in blood biochemistry parameters, including AST, ALT, urea, creatinine (Crea), IL‐6, and TNF‐α, between the DTGL group and the control group (Figure 7d–i). We collected gastrointestinal tract tissues and major organ tissues from mice treated for 7 and 14 days and performed hematoxylin and eosin (H&E) staining on tissue sections. The results showed that the tissue architecture in the DTGL‐treated group was comparable to that of the control group, with both groups exhibiting normal tissue morphology and no evidence of inflammatory cell infiltration or other abnormal phenomena (Figure S7, Supporting Information and Figure 7j,k). Additionally, DTGL was implanted into the livers of mice, and major organ tissues and blood samples were collected at 7 and 14 days post‐implantation. Biochemical analysis of the blood samples revealed no significant differences between the DTGL‐treated and control groups. Similarly, histological examination of the tissue sections did not reveal any abnormalities (Figure S8, Supporting Information). These findings collectively suggest that DTGL is well‐biocompatible and does not induce adverse inflammatory or tissue‐damaging effects in vivo.

## 3 Conclusion

In conclusion, we have developed an endoscopy‐deliverable, waxberry‐inspired, blue laser crosslinking “gel‐protein” dual‐network nano‐hemostatic material (DTGL) for GIB. The blue laser triggers GelMA to form an in situ network that adheres to and seals the wound, locking red blood cells in place as an initial physical barrier. Simultaneously, the loaded thrombin is released, inducing the conversion of fibrinogen in the blood into fibrin, which forms a second network, thereby enhancing clot stability. In vitro experiments demonstrated that the nanoscale hemostatic material achieved rapid blood coagulation within 5 s. The bionic waxberry‐like architecture, characterized by needle‐shaped macroporous channels, effectively preserved thrombin activity when exposed to the complex gastrointestinal environment. Simultaneously, the dual‐network reinforced blood clot exhibited enhanced structural stability, demonstrating significant potential for preventing rebleeding. This spider‐web‐like “gel‐protein” dual‐network hemostatic agent was successfully applied to bleeding models of mouse livers as well as the GIB (esophagus, stomach, and duodenum) of mini‐pigs, further verifying the hemostatic and adhesive properties of the nano‐hemostatic particles. The combination of endoscopy delivery, dual‐network formation, ultrafast hemostasis, and excellent biocompatibility makes DTGL a promising hemostatic agent for biomedical applications.

## 4 Experimental Section

### Materials

Thrombin (Thr) was obtained from Hunan Yige Pharmaceutical Co. Ltd. (Hunan, China). Cetyltrimethylammonium bromide (CTAB), 3‐Aminopropyltriethoxysilane (APTS), Tetraethyl orthosilicate (TEOS), gelatin methacryloyl (GelMA), Lithium phenyl (2,4,6‐trimethylbenzoyl) phosphinate (LAP) was purchased from Aladin Biotechnology Co. Ltd. (Shanghai, China). Poly(acrylic acid) (PAA) was purchased from Aladin Biotechnology Co. Ltd. (Shanghai, China). BCA protein assay kit was bought from Jianglai Biotechnology Co. Ltd. (Shanghai, China). Dullbecco's modified Eagle's medium (DMEM), fetal bovine serum, and trypsin were purchased from Gibco (Shanghai, China). Cell Counting Kit‐8 (CCK‐8) was purchased from Beijing Boxbio Science & Technology Co. Ltd (Beijing, China). Simulated gastric fluid and simulated intestinal fluid were obtained from Shenzhen Zhongtian Technology Co. Ltd. (Guangdong, China). APTT, PT, TT and Lacate Dehydrogenase (LDH) Activity assay kit was purchased from Solarbio Science & Technology Co. Ltd (Beijing, China). Human Thrombin/Antithrombin Complex (TAT) ELISA Kit was purchased from Solarbio Science & Technology Co. Ltd (Beijing, China). THROMBIN/FACTOR IIa Assay Kit was purchased from GENMED Scientifics Inc. (Boston, MA, USA). All other chemical reagents used in this experiment were of analytical grade and sourced from local suppliers.

### Synthesis of DMSN(+), DMSN(‐), MSN(+), MSN(‐)

Aminated dendritic mesoporous silica nanoparticles [DMSN(+)] were prepared according to a previous method.^[^ ^55^ ^]^ In brief, 0.8 mL ammonium hydroxide, 15 mL ether, and 5 mL ethanol were dissolved in 70 mL of deionized water. Then, 0.5 g of CTAB was incorporated into the emulsion. Subsequently, the mixture was magnetically stirred at 15 °C for 0.5 h with a stirring speed set at 1000 rpm. After stirring, TEOS (2.5 mL) and APTS (0.5 mL) were added to the mixture for an alkaline catalytic reaction lasting for 4 h. Then, concentrated hydrochloric acid (1 mL) was added to stop the reaction. Subsequently, the resulting solution was subjected to centrifugation at speeds ranging from 8000–10 000 rpm for 10 min to obtain white precipitate – DMSN(+) containing the template agent. This precipitate underwent three washes with anhydrous ethanol and ultra‐pure water, respectively prior to being dispersed in anhydrous ethanol for preservation. In order to remove the template agent from DMSN(+), a mixed solution of DMSN(+) and anhydrous ethanol (at a ratio of 1:8) was treated with concentrated hydrochloric acid. The resulting solution was transferred into a round‐bottomed flask placed on a heated magnetic stirrer where it was stirred at 1000 rpm for 24 h at 70 °C. Following this step, the product was divided into centrifuge tubes and subjected to high‐speed spinning at 8000–10 000 rpm for 10 min, resulting in the formation of white precipitate – DMSN(+). The obtained DMSN(+) washed thrice with ultra‐pure water before freeze‐drying. Next, 1 mg of DMSN(+) was mixed with 0.1 mL of poly(acrylic acid) (PAA) solution. Then, the resulting solution was centrifuged, washed three times with deionized water, and re‐dispersed in deionized water to obtain DMSN(‐).

Aminated mesoporous silica nanoparticles [MSN(+)] was synthesized as follows, 70 mL deionized water, 15 mL ethanol, and 0.8 mL ammonium hydroxide were mixed, followed by the addition of 0.5 g CTAB under magnetic stirring (800 rpm) at 35 °C for 30 min. Subsequently, 2.5 mL TEOS and 0.5 mL APTES were simultaneously added dropwise (0.5 mL/min) to the mixture, which was continuously stirred at 35 °C for 6 h. The reaction was terminated by adding 1 mL concentrated hydrochloric acid, and the product was centrifuged (8000–10 000 rpm, 10 min) to collect the MSN‐containing template. The precipitate was washed three times with anhydrous ethanol and ultrapure water, then dispersed in an ethanol‐HCl mixture (8:1 v/v) and refluxed at 60 °C with stirring (800 rpm) for 24 h to remove CTAB. Finally, the product was centrifuged, washed with ultrapure water, and freeze‐dried. Next, 1 mg of MSN (+) was mixed with 0.1 mL of PAA solution. Then, the resulting solution was centrifuged, washed three times with deionized water, and re‐dispersed in deionized water to obtain MSN (‐).

### Synthesis of DT

To load the particles with Thr, 1mg DMSN (+) was mixed with 1 mL of Thr (2 mg/mL) in DI water under sonication and then vortexized for 30 min. Then, the free Thr was removed by centrifugation. The obtained white precipitate was washed with pure water three times, and dispersed in ultrapure water or vacuum freeze‐dried into powder by a freeze‐dryer for future use.

### Thr Loading and Efficacy

To optimize the Thr ratio, DT were synthesized at weight ratios ranging from 0.125 to 4 mg of Thr per 1 mg of DMSN (+). The mass of Thr loaded in the DMSN was calculated by subtracting the mass of Thr in the supernatant from the initially added amount.

### Synthesis of DTGL

The freeze‐dried DT powder was dispersed in a GelMA prepolymer solution containing 10% m/v GelMA and 0.25% w/v LAP by ultrasound. Subsequently, the mixture was agitated at 37 °C for 1 h away from light. Finally, the mixed solutions were centrifuged (8000 rpm, 10 min) and washed three times with ultrapure water. The final pellet was collected and used for subsequent experiments.

### Characterizations

Transmission electron microscopy (TEM), and Scanning electron microscopy (SEM) were obtained using field emission transmission electron microscope (HT‐7700, Hitachi, Japan) and field emission scanning electron microscopy (Crossbeam 340, Zeiss, Germany), respectively. N_2_ adsorption/desorption and pore‐size distributions analysis were measured using a BSD‐PM1/2 apparatus. Zeta potentials of samples were determined by a Zetasizer Ultra (Malvern, UK). The absorbance of CCK‐8 was measured by the microplate reader of VICTOR Nivo 3S (PerkinElmer, UK).

### Viscosity Study

The viscosities of the DTGL solutions were evaluated using the following procedure: DTGL solutions, both with and without light irradiation, were prepared. A rotational rheometer (MCR302, Anton Paar, Austria) equipped with a 50 mm plate rotor (PP50) was employed for the viscosity measurements. The test parameters were configured as follows: a gap of 0.25 mm was maintained between the upper and lower plates, and the platform temperature was set to 37 °C. The shear rate was set as 0.1‐1000 rad s^−1^.

### In Vitro Biocompatibility

The NIH/3T3 (mouse embryonic fibroblasts), GES‐1 (human gastric mucosal epithelial cells) and HUVEC (human umbilical vein endothelial cells) were purchased from the cell bank of the Chinese Academy of Sciences. The cells were cultured in either nanoparticles‐conditioned medium or complete medium, and cell viability was assessed using the CCK‐8 cell proliferation/toxicity kit. The normal complete medium used was DMEM, which contained 10% fetal bovine serum and 1% penicillin/streptomycin. The conditioned medium was prepared by incubating 200 µL of the synthesized sample in 5 mL of complete medium for 24 h. The cells were seeded into a 96‐well cell culture plate at a density of 3000 cells per well. Cell viability was evaluated after incubation with either complete or conditioned medium for durations of 24, 48, and 72 h. Following addition of the CCK‐8 reagent, the cells were incubated at a temperature of 37 °C with a CO_2_ concentration maintained at 5% for a duration of 30 min before measuring absorbance at a wavelength of 450 nm.

### In Vivo Histocompatibility

To evaluate in vivo toxicity, mice were orally administered DTGL, with PBS‐treated mice serving as the control group. Weight changes in the mice were monitored. At predetermined intervals (2, 7, and 14 days), subjects were euthanized for venous blood collection and subsequent biochemical profiling, including analysis of AST, ALT, UREA, CREA, IL‐6, and TNF‐α. Major organs (heart, liver, spleen, lungs, kidneys) and gastrointestinal tissues were harvested for histological examination through hematoxylin‐eosin (H&E) staining. In a parallel hepatic implantation model, DTGL was surgically grafted onto murine liver surfaces. Systemic toxicity and tissue responses were similarly assessed through blood chemistry profiling and histopathological evaluation at 7 and 14 days post‐implantation.

### Hemostatic Performance of the DTGL In Vitro—Clotting Time Assay

The clotting time assay was conducted following a previously established protocol.^[^ ^56^ ^]^ In brief, the anticoagulant whole blood was re‐calcified by adding 0.1 M calcium chloride at a volume ratio of 10:1 and mixed evenly for 10 s. Then, 50 µL of the activated blood was dispensed into each well of a 96‐well plate. Next, each group of sample solutions (Ca^2+^, DMSN, DGL, Thr, FG DT, DTGL) was immediately introduced, followed by light illumination if deemed necessary. At defined time points, the plate was rinsed with normal saline to terminate the coagulation reaction and the fluid was promptly aspirated and thoroughly washed until the solution became clear. The clotting time was recorded upon the formation of a uniform clot in the well. Subsequently, the samples were fixed in 2% glutaraldehyde for 12 h and then sequentially dehydrated using ethanol and tert‐butanol gradients before being sputter‐coated with gold and observed under SEM.

### Clotting kinetics

Fifty microliters of sample solutions were added to the bottom of individual wells in a 24‐well plate. Then, 50 µL of whole blood was introduced into the same plate and mixed with the samples followed by immediate utilization of a blue laser if necessary. At each designated time point, 2.9 mL distilled water was added to each well and maintained for 10 min to lyse the erythrocytes that were not entrapped within the blood clots. The erythrocytes were hemolyzed, and the absorbance of the resulting supernatant was measured by microplate reader (Multiskan GO 1510, Thermo Fisher Scientific, USA)) at 540 nm. The blood sample mixed with normal saline served as an experimental control. Three replicates were conducted for each sample.

### APTT Assay

The activated partial thromboplastin time (APTT) assay was employed to evaluate the effects of materials on the intrinsic coagulation pathway. First, sodium citrate‐anticoagulated whole blood was centrifuged at 3000 rpm for 10 min to prepare platelet‐poor plasma (PPP). Subsequently, 20 µL of test materials (Ca^2+^, DMSN, DGL, Thr, FG, DT, and DTGL) were mixed with 50 µL PPP respectively. Finally, coagulation time was measured following the manufacturer's protocol of the APTT assay kit, with clotting time recorded for each sample.

### PT Assay

The prothrombin time (PT) assay was utilized to assess the effects of materials on the extrinsic coagulation pathway. Each test material (Ca^2+^, DMSN, DGL, Thr, FG, DT, and DTGL) was mixed with 50 µL platelet‐poor plasma (PPP) at a volume of 20 µL. Coagulation time was then measured according to the PT assay kit protocol, with clotting time recorded for all experimental groups.

### TT Assay

The thrombin time (TT) assay was employed to evaluate the effects of materials on the common coagulation pathway. Each test material (Ca^2+^, DMSN, DGL, Thr, FG, DT, and DTGL) was mixed with 50 µL platelet‐poor plasma (PPP) at a volume of 20 µL. Coagulation time was subsequently measured following the TT assay kit protocol, with clotting time recorded for all experimental groups.

### Blood Cell and Platelet Adhesion

About 20 µL each of Ca^2+^, DMSN, DGL, Thr, FG, DT, and DTGL were placed into a 24‐well plate. Then, 50 µL of whole blood was added to each well. The samples were incubated for 7 min, with light illumination if necessary. After incubation, each sample was washed three times with PBS to remove non‐adherent red blood cells (RBCs). The adherent RBCs were lysed with 2 mL of deionized (DI) water to release hemoglobin. Next, 100 µL of the supernatant was transferred to a 96‐well plate, and the absorbance was measured at 562 nm using a microplate reader. As a reference, the absorbance of a mixture of 50 µL RBC suspension and 2 mL DI water was measured. The percentage of adherent RBCs was calculated using the following equation:

(1) $$\mathrm{Percentage}\mathrm{of}\mathrm{red}\mathrm{blood}\mathrm{cell}=\left(OD_{\mathrm{sample}}/OD_{\mathrm{reference}}\right)\times 100\%$$

For platelet adhesion experiments, platelet‐rich plasma (PRP), donated by a blood center, was used. A volume of 50 µL PRP was added to each sample group as described above, mixed thoroughly, and co‐incubated at 37 °C for 10 min. The samples were rinsed three times with PBS to remove non‐adherent platelets and then treated with 1% Triton X‐100 to lyse the platelets and release lactate dehydrogenase (LDH). The concentration of LDH was measured using a Lactate Dehydrogenase Assay Kit. The absorbance of the supernatant from each sample was measured at 450 nm (referred to as OD _sample_). The absorbance of PRP not exposed to hemostatic materials was measured at 490 nm as a reference. The percentage of adherent platelets was calculated using the following equation:

(2) $$\mathrm{Percentage}\mathrm{of}\mathrm{adherent}\mathrm{platelets}=\left(OD_{\mathrm{sample}}/OD_{\mathrm{reference}}\right)\times 100\%$$

### Thrombin Activity Test

Samples from different groups were pre‐treated with digestive fluids (simulated gastric fluid, simulated intestinal fluid, real gastric fluid, or real intestinal fluid) for a specified duration. After treatment, the digestive fluids were removed, and the samples were resuspended in pH 7.0 phosphate‐buffered saline (PBS) to form sample solutions. The activity of each sample solution was assessed using a blood thrombin activity assay kit. Subsequently, 50 µL of each sample solution was transferred to centrifuge tubes containing 100 µL of anticoagulated blood. After 30 s, the centrifuge tubes were placed in an inverted position on a flat surface, and the coagulation effect was observed. Serum was collected from each tube to analyze changes in TAT (thrombin‐antithrombin complex) and FIB (fibrinogen) levels.

### Clot Stability Test

Different sample solutions were added to 100 µL of blood to initiate and promote clotting, respectively, crosslinking with a blue laser if necessary. After 30 s, the tubes were inverted on the table to confirm clot formation. Then, 300 µL of SGF, SIF, real gastric fluid, or real intestinal fluidsolution was added to soak the clots for varying incubation times. Next, the clots were extracted for subsequent measurements of weight and volume. Additionally, the supernatants were collected to meassure OD 540 nm.^[^ ^20^, ^57^ ^]^

### In Vivo Liver Hemostatic Ability

All animal studies were approved by the Institutional Animal Care and Use Committee of the Third Military Medical University (Animal Ethical Statement Number: AMUWEC20235153). All experimental animals were purchased from the Experimental Animal Center of the Third Military Medical University. A mouse hemorrhage liver model (male BALB/C, 8–12 weeks) to investigate the in vivo hemostatic ability of DTGL was used. Initially, mice were anesthetized by 1% pentobarbitone. Preweighed filter paper was positioned beneath the liver. Then, one‐quarter of the liver was cuted off to induce bleeding using scissors. Immediately after, 100 µL of hemostatic agents (Thr, DT, DTGL) were applied to the bleeding site with/without a 30‐second blue laser irradiation. The weight of blood‐soaked filter paper, extent of bleeding area, and the hemostatic time were measured. After euthanizing the mice, their livers were excised and fixed for subsequent observation under SEM.

### Gastrointestinal Hemorrhage Model

Miniature pigs weighing 15–20 kg (male) were utilized. Anesthesia and monitoring during the endoscopy operation procedures were carried out in accordance with previously established protocols.^[^ ^58^, ^59^, ^60^, ^61^ ^]^ The GIB models were created under endoscopy using biopsy forceps to selectively target vascular‐rich regions in the esophagus, stomach, and duodenum. Until the bleeding model was successfully established, different hemostatic agents were delivered through a catheter and sprayed onto the bleeding site. In case of DTGL, immediate activation of the BLE was performed for photocrosslinking. Subsequently, wounds were observed for 2 min and rinsed with normal saline to assess blood clot loss and rebleeding. Pigs were monitored daily after resuscitation for mental state as well as hematemesis, melena, and any signs of rebleeding. After 48 h, pigs were anesthetized and rescoped to visualize the model sites and check for any rebleeding. Tissue samples from treated sites were harvested for SEM analysis.

### Statistical Analysis

All data were obtained from at least 3 replicate samples and presented as means ± S.D. Normality of distributions was analyzed by Shapiro‐Wilk test. Independent t test and one‐way ANOVA analysis of variance with Tukey post‐hoc analysis was performed for comparison of two or multiple groups respectively. Statistical analyses were conducted using GraphPad Prism 9.0.0, and a two‐sided *p* value < 0.05 was considered statistically significant. *p < 0.05, **p < 0.01, ***p < 0.001, ****p < 0.0001.

### Ethics Approval Statement

All procedures were approved by the Medical Ethics Committee of Xinqiao Hospital (2023‐159‐01). Informed consent was acquired from the blood donors. All animal experimental protocols were approved by the Laboratory Animal Welfare and Ethics Committee of the Army Medical University (Animal Ethical Statement Number: AMUWEC20235153).

## Conflict of Interest

The authors declare no conflict of interest.

## Supporting information



Supporting Information

Supplemental Movie 1

Supplemental Movie 2

Supplemental Movie 3

Supplemental Movie 4

Supplemental Movie 5

Supplemental Movie 6

Supplemental Movie 7

Supplemental Movie 8

Supplemental Movie 9

Supplemental Movie 10

Supplemental Movie 11

## Data Availability

The data that support the findings of this study are available from the corresponding author upon reasonable request.
