# Supplementary material for: Bioinspired Smart Nanogels for Rapid Blue Laser‐Activated Hemostasis in Gastrointestinal Bleeding
Source: Adv Mater. 2025 Jun 4;37(38):2506955. doi: 10.1002/adma.202506955 (PMC12464633; doi:10.1002/adma.202506955)
Supplement: Supplementary file 1 — Supporting Information [file ADMA-37-2506955-s008.docx]

**Table S1**. Gas sorption parameters of DMSN (+), DMSN (-), MSN (+), and MSN (-).

|  | SBET (m^2^ g^-1^) | Pore volume (cm^3^ g^-1^) |
| --- | --- | --- |
| DMSN (+) | 668.2 | 1.2 |
| DMSN (-) | 659.5 | 1.2 |
| MSN (+) | 385.3 | 0.8 |
| MSN (-) | 375.0 | 0.8 |


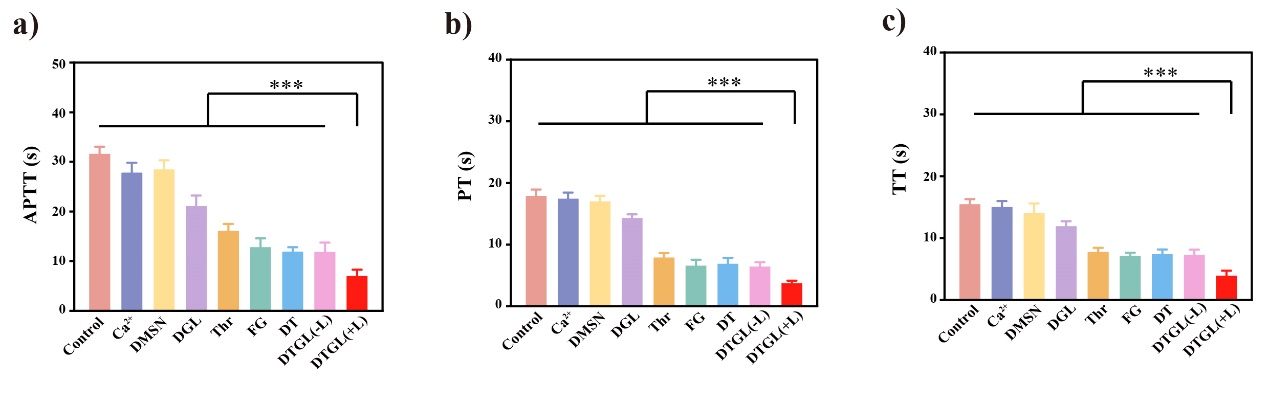


**Figure S1.** The activated partial thromboplastin time (APTT) (a) and prothrombin time (PT) (b) and thrombin time (TT) (c) analysis (n = 5). Data are presented as mean ± S.D. ****p* < 0.001.


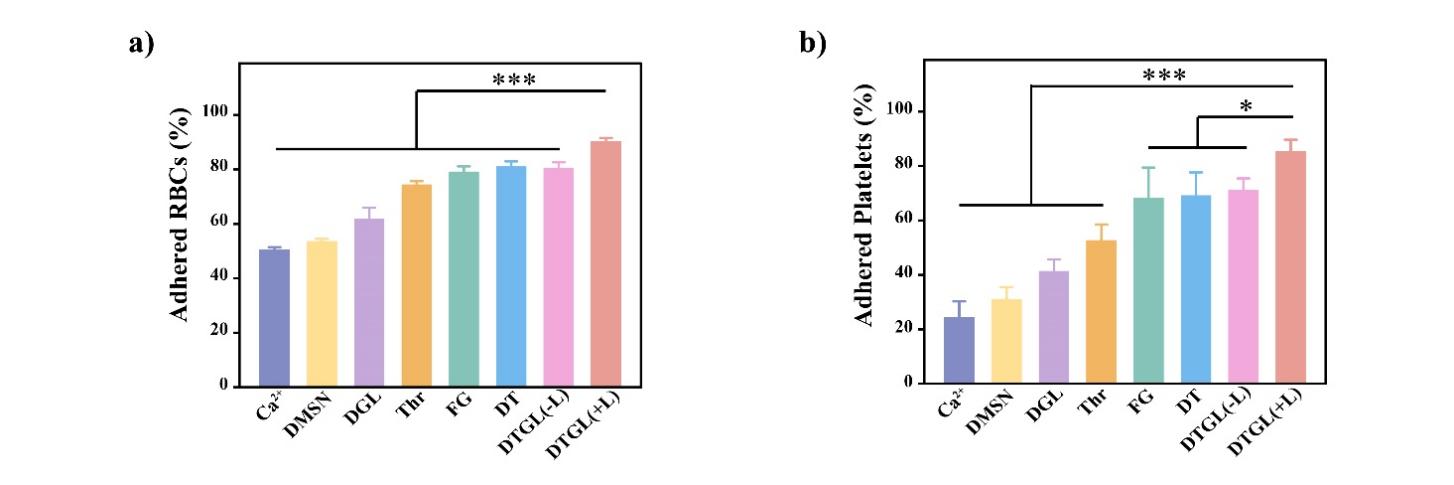


**Figure S2.** a) The percentage of adhered RBCs (n = 3). b) Percentage of adhered platelets (n = 3). Data are presented as mean ± S.D. **p* < 0.05, ****p* < 0.001.


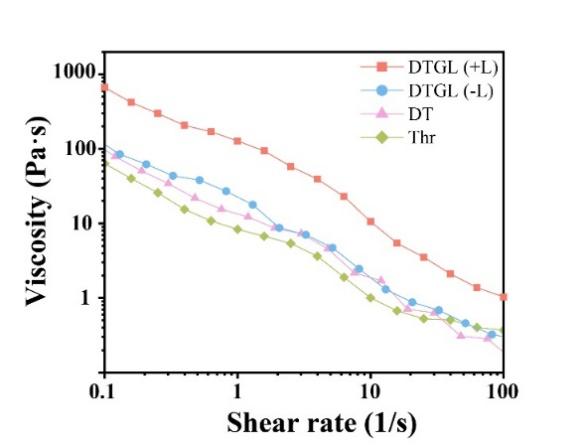


**Figure S3.** Viscosity analysis of blood clots under different conditions: Thrombin (Thr), DT, DTGL(-L), and DTGL(+L).


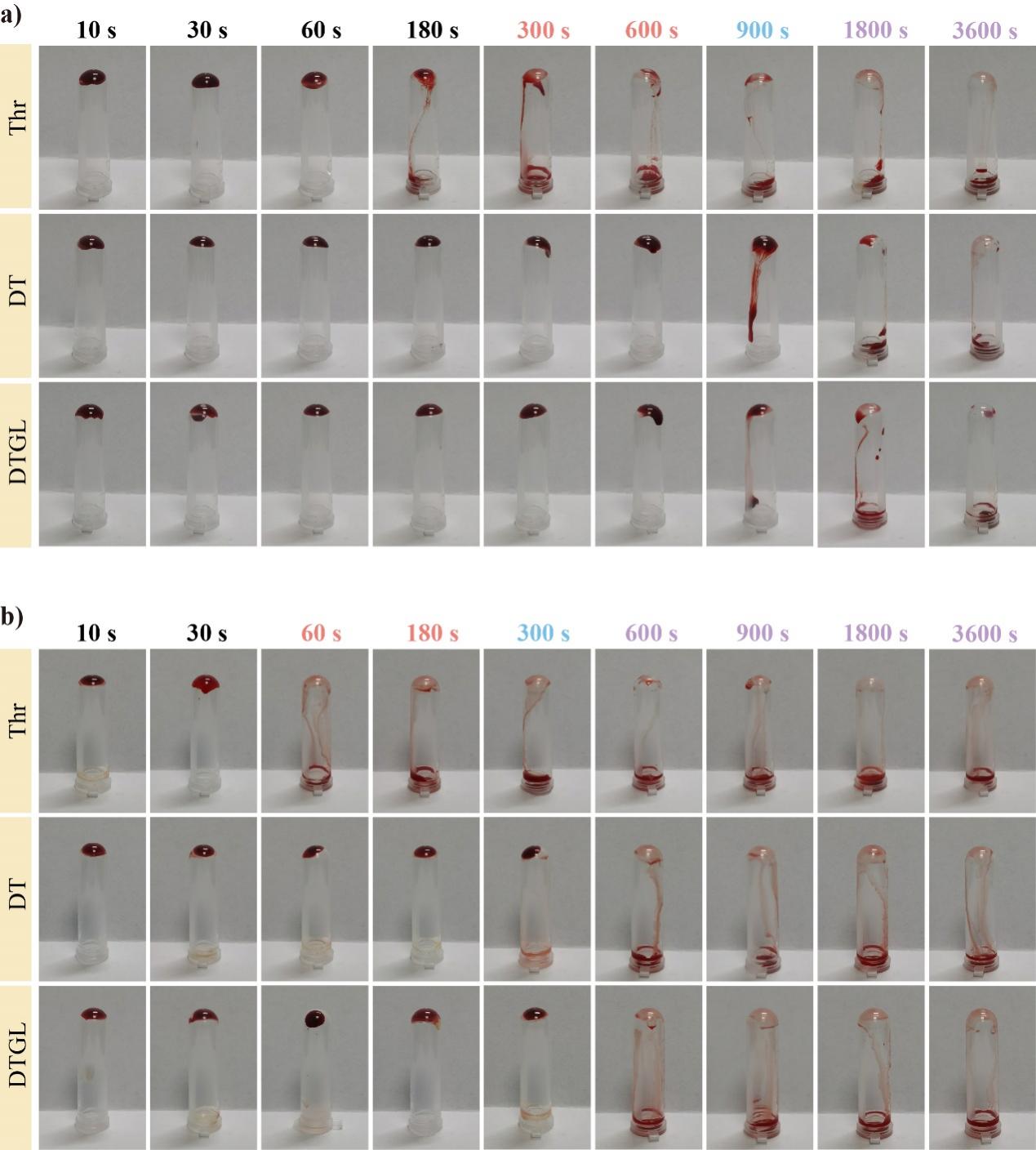


**Figure S4.** Photographs of blood coagulation effect in (a) simulated gastric fluid (SGF) and (b) real gastric fluid (GF) for Thr, DT, and DTGL over 3600 seconds.


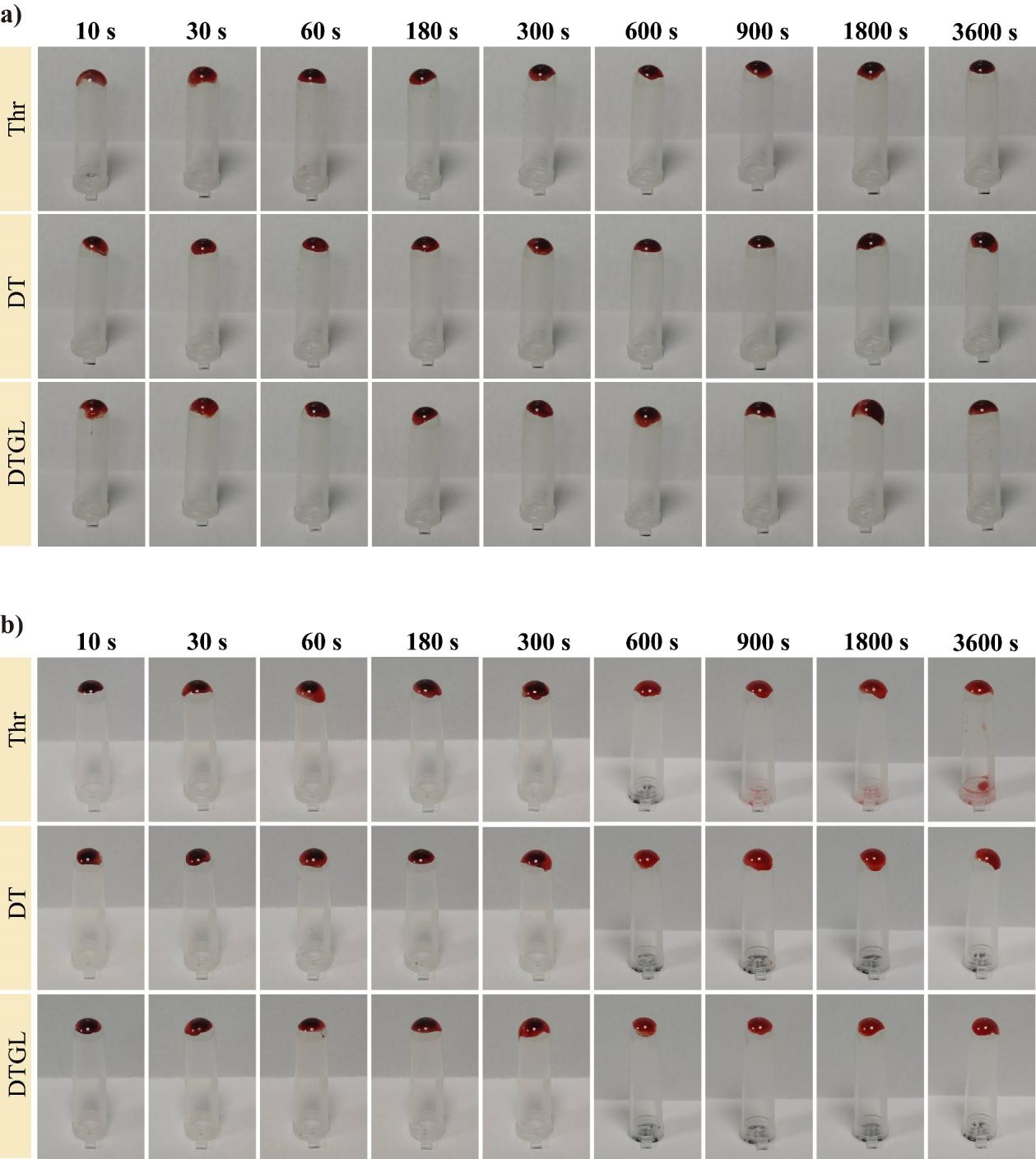


**Figure S5.** Photographs of blood coagulation effect in (a) simulated intestinal fluid (SIF) and (b) real intestinal fluid (IF) for Thr, DT, and DTGL over 3600 seconds.


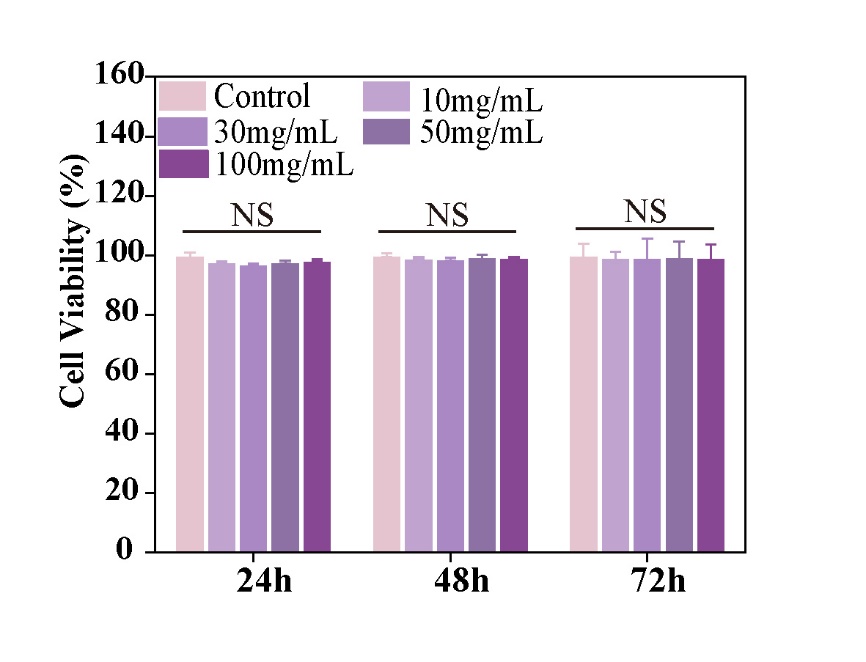


**Figure S6.** Cytotoxicity of different concentrations of DTGL (10, 30, 50, 100 mg/mL) on 3t3 cells after 24, 48, and 72 hours of culture (n = 3). Data are presented as mean ± S.D. NS, no significant difference.


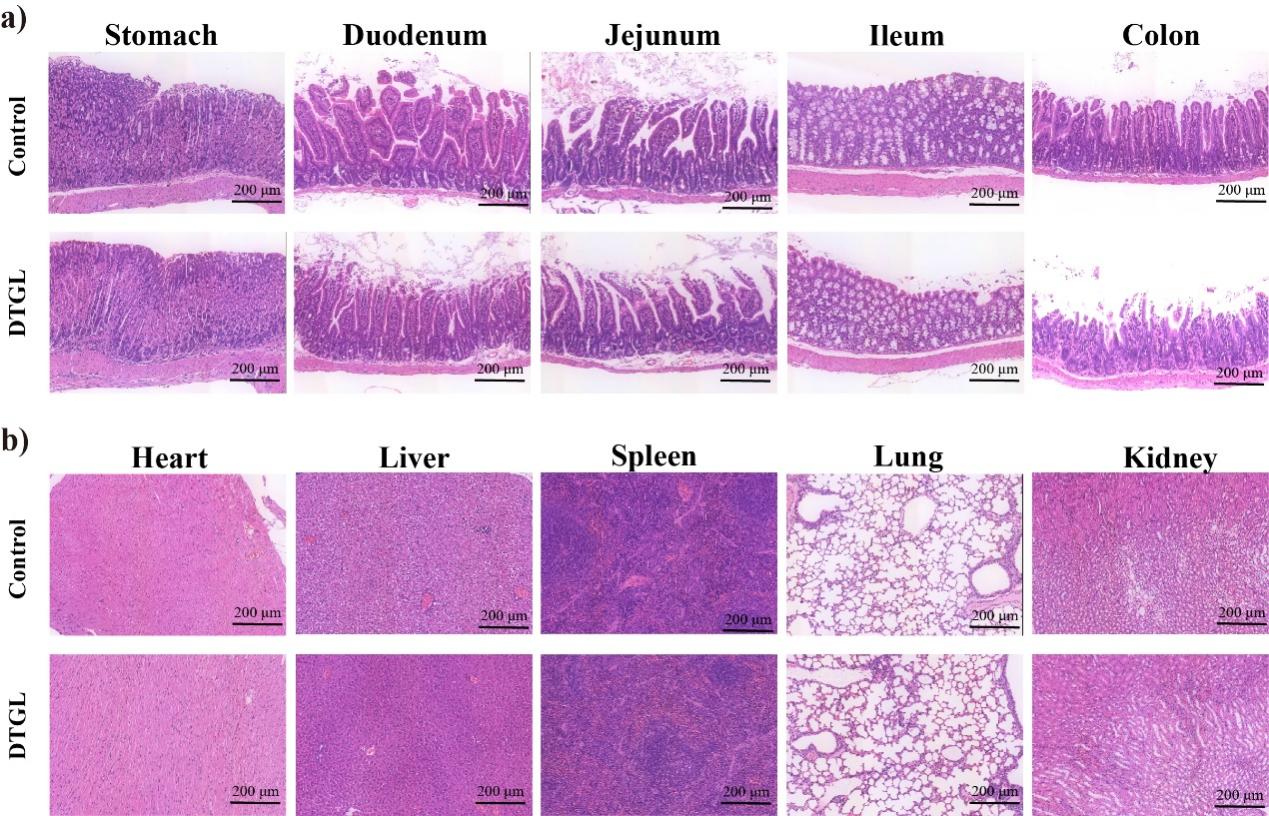


**Figure S7.** H&E staining of the gastrointestinal tract (stomach, duodenum, jejunum, Ileum, Colon) (a) and major organ tissues (heart, liver, spleen, lung, kidney) (b) on 7 days for intragastric administration.


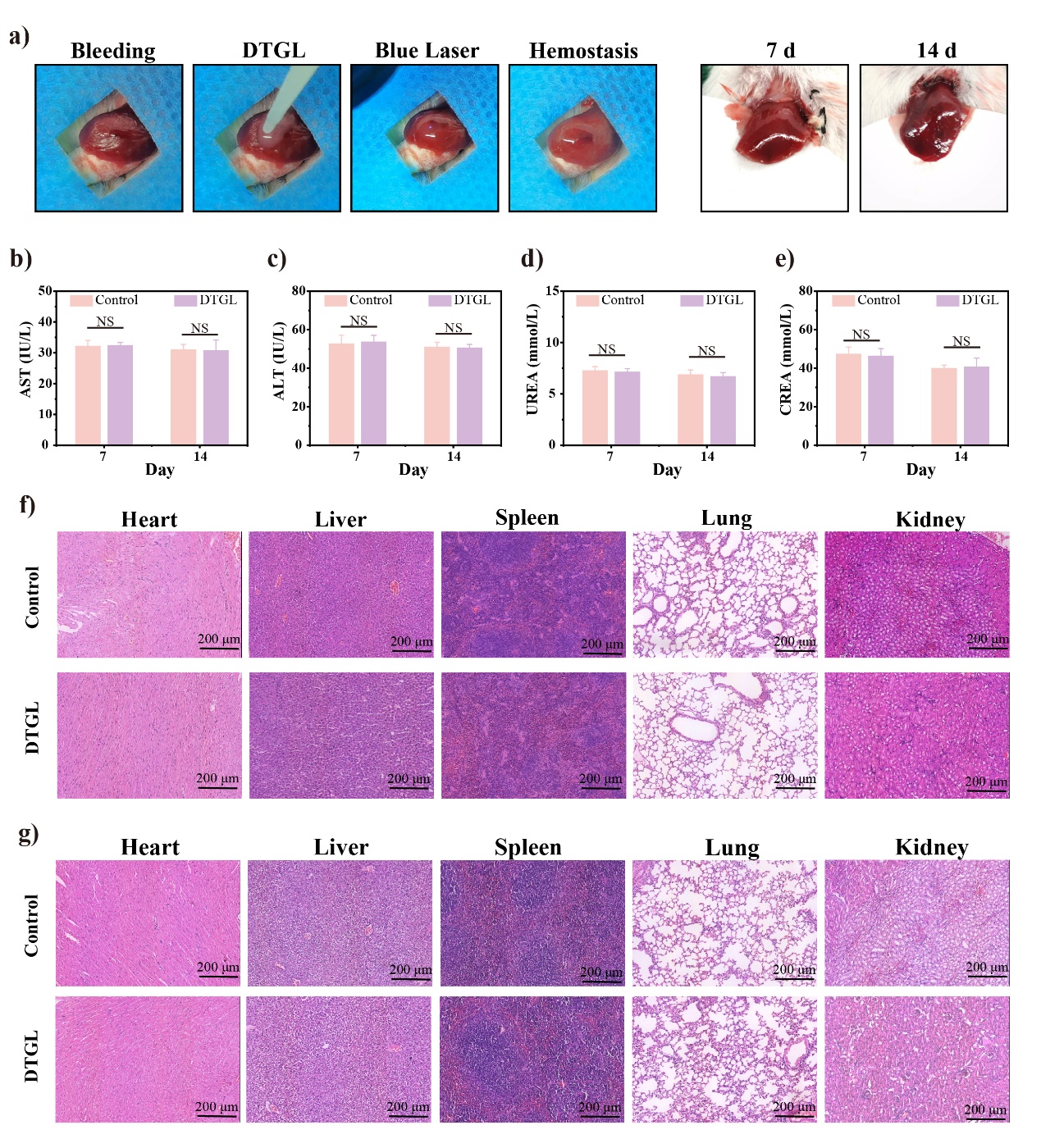


**Figure S8. In vivo biocompatibility evaluation of DTGL hepatic implantation.** (a) Photographs of DTGL implantation in mice liver. (b-e) Blood biochemical analysis at postoperative day 7 and 14. (f,g) Histopathological assessment of major organs (heart, liver, spleen, lungs, kidneys) following DTGL implanted at day 7 (f) and 14 (g) through H&E staining.

Movie S1. Mouse hepatic lobectomy model without any intervention.

Movie S2. Hemostatic effect of Thr on mouse hepatic lobectomy model.

Movie S3. Hemostatic effect of DT on mouse hepatic lobectomy model.

Movie S4. Hemostatic effect of DTGL (-L) on mouse hepatic lobectomy model.

Movie S5. Hemostatic effect of DTGL (+L) on mouse hepatic lobectomy model.

Movie S6. Esophagus hemostasis by DTGL under BLE in porcine GIB model.

Movie S7. Esophagus hemostasis by Thr under BLE in porcine GIB model.

Movie S8. Stomach hemostasis by DTGL under BLE in porcine GIB model.

Movie S9. Stomach hemostasis by Thr under BLE in porcine GIB model.

Movie S10. Duodenum hemostasis by DTGL under BLE in porcine GIB model.

Movie S11. Duodenum hemostasis by Thr under BLE in porcine GIB model.
